# A Poly(Lactic-*co*-Glycolic) Acid Nanovaccine Based on Chimeric Peptides from Different *Leishmania infantum* Proteins Induces Dendritic Cells Maturation and Promotes Peptide-Specific IFNγ-Producing CD8^+^ T Cells Essential for the Protection against Experimental Visceral Leishmaniasis

**DOI:** 10.3389/fimmu.2017.00684

**Published:** 2017-06-13

**Authors:** Evita Athanasiou, Maria Agallou, Spyros Tastsoglou, Olga Kammona, Artemis Hatzigeorgiou, Costas Kiparissides, Evdokia Karagouni

**Affiliations:** ^1^Laboratory of Cellular Immunology, Department of Microbiology, Hellenic Pasteur Institute, Athens, Greece; ^2^DIANA-Lab, Hellenic Pasteur Institute, Athens, Greece; ^3^Laboratory of Polymer Reaction Engineering, Chemical Process and Energy Resources Institute, Centre for Research and Technology-Hellas, Thessaloniki, Greece; ^4^Laboratory of Chemical Engineering B, Department of Chemical Engineering, Aristotle University of Thessaloniki, Thessaloniki, Greece

**Keywords:** *Leishmania*, chimeric peptides, peptide-based vaccine, poly(lactic-*co*-glycolic) acid nanoparticles, dendritic cell transcriptome

## Abstract

Visceral leishmaniasis, caused by *Leishmania* (*L*.) *donovani* and *L. infantum* protozoan parasites, can provoke overwhelming and protracted epidemics, with high case-fatality rates. An effective vaccine against the disease must rely on the generation of a strong and long-lasting T cell immunity, mediated by CD4^+^ T_H1_ and CD8^+^ T cells. Multi-epitope peptide-based vaccine development is manifesting as the new era of vaccination strategies against *Leishmania* infection. In this study, we designed chimeric peptides containing HLA-restricted epitopes from three immunogenic *L. infantum* proteins (cysteine peptidase A, histone H1, and kinetoplastid membrane protein 11), in order to be encapsulated in poly(lactic-*co*-glycolic) acid nanoparticles with or without the adjuvant monophosphoryl lipid A (MPLA) or surface modification with an octapeptide targeting the tumor necrosis factor receptor II. We aimed to construct differentially functionalized peptide-based nanovaccine candidates and investigate their capacity to stimulate the immunomodulatory properties of dendritic cells (DCs), which are critical regulators of adaptive immunity generated upon vaccination. According to our results, DCs stimulation with the peptide-based nanovaccine candidates with MPLA incorporation or surface modification induced an enhanced maturation profile with prominent IL-12 production, promoting allogeneic T cell proliferation and intracellular production of IFNγ by CD4^+^ and CD8^+^ T cell subsets. In addition, DCs stimulated with the peptide-based nanovaccine candidate with MPLA incorporation exhibited a robust transcriptional activation, characterized by upregulated genes indicative of vaccine-driven DCs differentiation toward type 1 phenotype. Immunization of HLA A2.1 transgenic mice with this peptide-based nanovaccine candidate induced peptide-specific IFNγ-producing CD8^+^ T cells and conferred significant protection against *L. infantum* infection. Concluding, our findings supported that encapsulation of more than one chimeric multi-epitope peptides from different immunogenic *L. infantum* proteins in a proper biocompatible delivery system with the right adjuvant is considered as an improved promising approach for the development of a vaccine against VL.

## Introduction

Leishmaniasis, a group of vector-borne parasitic diseases caused by dimorphic protozoan flagellates of the genus *Leishmania*, is highly diverse and complex with a wide spectrum of clinical forms in humans, ranging from the self-healing cutaneous leishmaniasis (CL) to the potentially fatal visceral leishmaniasis (VL). With an estimated incidence of 0.3 million cases and over than 30,000 deaths per year, VL has emerged as a serious global problem and important public health concern with major clinical and socioeconomic impacts (1, 2). In South Europe, Central, and South America, VL is caused by *Leishmania (L.) infantum* (synonym *L. chagasi*) and is transmitted as a zoonosis with the domestic dog serving as the main reservoir of the parasite, especially in the urban and suburban areas (3).

Current control tactics for VL rely on chemotherapy to alleviate the disease and on vector control to reduce transmission. Since the arsenal of drugs available is limited and chemotherapy gathers many disadvantages with most prominent the toxicity and the emergence of resistance, the development of a prophylactic, safe, and cost affordable vaccine is considered high priority. The success of vaccine development depends on understanding the immunology of host–pathogen interactions, choosing appropriate antigenic candidates, and selecting the right adjuvant and/or delivery vehicle.

It is well established that CD4^+^ T helper type 1 (T_H1_) cells are critical for the control of *L. infantum* infection owing to their ability to produce IFNγ, which activates macrophages to kill parasites *via* nitric oxide (NO) production leading to reduction in parasitic burden (4, 5). However, nowadays, it is clear that CD8^+^ T cells also play an important role in the mechanisms involved in cure of and resistance to VL, either by production of IFNγ and macrophage activation or by direct killing of parasitized macrophages, or *via* a combination of both effects (6, 7). Thus, an effective vaccine against the disease must rely on the generation of a strong and long-lasting T cell immunity (7).

Almost a decade ago, T cell epitope prediction *via* bioinformatics analysis of protein sequences has been proposed as an alternative for rational vaccine development (8). Recent immunoinformatics approaches utilize multiple algorithms for predicting epitopes, HLA-binding, transporter of antigen processing (TAP) affinity, proteasomal cleavage, etc., in order to explore the use of peptide epitopes with the highest probability of inducing protective immune responses (9). Such bioinformatics tools predict promiscuous epitopes presented by different HLA supertypes, providing a way to surmount the obstacle of HLA heterogeneity in human populations through the design of “polytope vaccines” against several pathogens. Although an ideal “polytope vaccine” for human population seems to be still difficult to achieve, several research groups have studied the protective potential of epitope vaccines against *Leishmania* infectious challenges in experimental models (10, 11). Peptide-based vaccines offer considerable advantages over other vaccine types, such as cost-effective production, safety, stability under different conditions, high specificity due to defined epitopes, and decreased chance of stimulating a response against self-antigens. On the other hand, they have drawbacks including low immunogenicity and rapid degradation by endopeptidase or exopeptidase activity in the injection site or in circulation. Thus, peptides need to be combined with delivery systems and/or adjuvants such as immune modulators in order to properly activate the innate and adaptive arms of the immune system (12).

Several studies have indicated that peptide-based vaccines may benefit from particulate delivery systems that mimic the size and structure of a pathogen, facilitating uptake by dendritic cells (DCs) and increasing the probability of peptide cross-presentation (13–15). DCs are the most proficient antigen-presenting cells in capturing, processing, and presenting antigens, as well as triggering T cell responses. Further, they exclusively own the capacity of primary activating naïve T lymphocytes. Classically, extracellular antigens are taken up by DCs, processed into short peptides, and presented on major histocompatibility complex (MHC) class II molecules to activate CD4^+^ T cells. However, intracellular phagocytosis of exogenous antigens mediated by nanoparticles (NPs) can dramatically enhance cross-presentation, where the antigen is processed in the cytoplasm for presentation on MHC class I molecules activating CD8^+^ T cell responses (16, 17). Among particulate delivery systems, poly(lactic-*co*-glycolic) acid (PLGA) polymers have received considerable interests in recent years for potent use in antigen delivery, due to the numerous advantages they provide. Commercially available at different molecular weights (MW) and co-polymer compositions, PLGA polymers are biodegradable and biocompatible, protect the encapsulated molecules from degradation, allow co-encapsulation of antigens and immune modulators in the same particle, offer the possibility of sustained release, can undergo surface modification, and be targeted to antigen-presenting cells, while their particulate nature can increase uptake and cross-presentation (18, 19). PLGA NPs have already been tested as antigen-delivery systems in different experimental models toward the development of an effective vaccine against leishmaniasis with encouraging results (20–22).

In the process of vaccine development against infectious diseases, there is often a strong need to monitor a great number of compounds for their immunogenicity. A functional high-throughput screen of candidate vaccines can be carried out to test their ability to activate innate immune cells *in vitro*, since such assays could be predictive of *in vivo* immunogenicity. DCs play pivotal role in the induction of adaptive immunity priming naïve T cells, and, consequently, in orchestration of immune responses upon vaccination. Thus, *in vitro* assays monitoring DCs activation after stimulation represent a robust biological platform to predict the immunological potential of novel vaccine compounds and, therefore, could be considered as a tool for the preclinical assessment of their immunogenicity (23, 24). Moreover, recently, the scientific community has focused its interest on the definition of transcriptional signatures to study immune responses induced by already existing and candidate vaccines (25, 26). Data obtained from the gene-expression profile of DCs stimulated with different antigens, adjuvants, antigen-delivery systems, or candidate vaccines may guide the development of an improved vaccination strategy (24, 27, 28).

In this study, we designed synthetic long peptides (chimeric peptides) using proper amino acid (aa) linkers and multi-epitope peptides containing HLA class I-restricted epitopes of the *L. infantum* proteins cysteine peptidase A (CPA), histone H1, and kinetoplastid membrane protein 11 (KMP-11). Each chimeric peptide was encapsulated in PLGA NPs alone or in combination with the adjuvant monophosphoryl lipid A (MPLA), or in PLGA NPs surface modified with an octapeptide (p8) mimicking the TNFα-docking region with tumor necrosis factor receptor II (TNFRII), with the view of constructing experimental peptide-based nanovaccines. In the context of a cell-based preclinical system, we aimed to compare the capacity of the differentially functionalized nanovaccine candidates to stimulate the immunomodulatory properties of DCs, which are critical regulators of vaccine-induced immunity, in order to select the most promising for *in vivo* evaluation. To that end, the expression of activation markers, the production of cytokines, the ability to stimulate allogeneic T cells, and the gene-expression profile of bone marrow-derived DCs from HLA A2.1 transgenic mice were examined. From the *in vitro* screening, the mix of PLGA nanoformulations with MPLA incorporation emerged as a promising peptide-based nanovaccine and thus, its ability to promote antigen-specific CD8^+^ T cell responses and to confer protection against *L. infantum* infection was evaluated in HLA A2.1 transgenic mice, a strain that enables the modeling of human T cell immune responses to HLA A*0201-restricted epitopes.

## Materials and Methods

### Chimeric Peptide Design and Synthesis

Two multi-epitope peptides of CPA (CPA_p2: 160-189 aa and CPA_p3: 273-302 aa), two multi-epitope peptides of histone H1 (H1_p1: 1-20 aa and H1_p3: 43-61 aa), and one multi-epitope peptide of KMP-11 (KMP-11_p1: 4-23 aa), derived from a previously described *in silico* analysis of the three *L. infantum* proteins (29) and designed in a way to contain HLA-restricted epitopes (Table 1), formed the basis for the design of chimeric peptides (one for each protein) with the use of proper linkers. HLA A*0201-restricted epitopes derived from the *L. infantum* proteins and included in the chimeric peptides were predicted using the online available algorithms SYFPEITHI (30) and EpiJen (31) with a cutoff score adjusted to ≥18 and <50 nM, respectively (Table 2). SYFPEITHI was also used for the *in silico* prediction of potent H2-Kb- or H2-Db-restricted epitopes (Table 2). The chimeric peptides (chCPAp, chH1p, and chKMP-11p) were synthesized by GeneCust (Labbx, Dudelange, Luxembourg) with ≥95% purity. Synthetic chimeric peptides were dissolved in DMSO or dH_2_O, according to their hydrophobicity, by vigorous pipetting and stored at −80°C in aliquots until use.

**Table 1:** Chimeric peptides containing multi-epitope peptides of the *Leishmania infantum* immunogenic proteins cysteine peptidase A (CPA), histone H1, and kinetoplastid membrane protein 11 (KMP-11).

Name	Sequence (${\text{NH}}_{2^{-}}$ … -COOH)	Multi-epitope peptide	HLA supertypes	Linker	Length (aa)	Molecular weights (Da)
chCPAp	GNIEGQWALKNHSLVSLSEQVLVSCDNIDD**AAY**LYFGGVVTLCFGLSLNHGVLVVGFNRQAKP	CPA_p2 and CPA_p3	HLA-A2 (A*0201)	AAY	65	6,778.77
			HLA-A3 (A*03)			
			HLA-A24 (A*2402)			
			HLA-DRB1			
			HLA-DPA1			
			HLA-DQA1			

chH1p	MSSDSAVAALSAAMTSPQKS**AAY**AGAKKAGAKKAVRKVATPKK	H1_p1 and H1_p3	HLA-A2 (A*0201)	AAY	43	4,236.02
			HLA-A3 (A*03)			
			HLA-DRB1			
			HLA-DQA1			

chKMP-11p	AKFVAAWTLKAAA**HEYGAEALERAG**TYEEFSAKLDRLDEEFNRKM	PADRE and KMP-11_p1	HLA-A2 (A*0201)	HEYGAEALERAG	45	5,135.79
			HLA-A3 (A*03)			
			HLA-A24 (A*2402)			
			HLA-DRB1			
			HLA-DPA1			
			HLA-DQA1			

**Table 2:** HLA A*0201, H2-Db, and H2-Kb restricted epitopes included in the chimeric peptides from the *Leishmania infantum* immunogenic proteins cysteine peptidase A (CPA), histone H1, and kinetoplastid membrane protein 11 (KMP-11).

Name	Sequence (${\text{NH}}_{2^{-}}$ … -COOH)	HLA A*0201-restricted epitopes	H2-Db restricted epitopes	H2-Kb restricted epitopes
chCPAp	GNIEGQWALKNHSLVSLSEQVLVSCDNIDD**AAY**LYFGGVVTLCFGLSLNHGVLVVGFNRQAKP	*Nonamers*	*Nonamers*	No predicted epitopes
		160-GNIEGQWAL-168	166-WALKNHSLV-174	
		168-LKNHSLVSL-176	284-GLSLNHGVL-292	
		172-SLVSLSEQV-180	*Decamers*	
		175-SLSEQVLVS-183	276-GGVVTLCFGL-285	
		273-LYFGGVVTL-281	284-GLSLNHGVLV-293	
		277-GVVTLCFGL-285		
		279-VTLCFGLSL-287		
		283-FGLSLNHGV-291		
		284-GLSLNHGVL-292		
		286-SLNHGVLVV-294		
		*Decamers*		
		167-ALKNHSLVSL-176		
		172-SLVSLSEQVL-181		
		173-LVSLSEQVLV-182		
		175-SLSEQVLVSC-184		
		284-GLSLNHGVLV-293		
		285-LSLNHGVLVV-294		
		286-SLNHGVLVVG-295		

chH1p	MSSDSAVAALSAAMTSPQKS**AAY**AGAKKAGAKKAVRKVATPKK	*Nonamers*	*Decamers*	No predicted epitopes
		2-SSDSAVAAL-10	5-SAVAALSAAM-14	
		49- GAKKAVRKV-57		
		*Decamers*		
		1-MSSDSAVAAL-10		
		48-AGAKKAVRKV-57		

chKMP-11p	AKFVAAWTLKAAA**HEYGAEALERAG**TYEEFSAKLDRLDEEFNRKM	*Nonamers*	*Decamers*	No predicted epitopes
		2-ATTYEEFSA-10	10-AKLDRLDEEF-19	
		11-KLDRLDEEF-19		
		*Decamers*		
		3-TTYEEFSAKL-12		
		14-RLDEEFNRKM-23		

*The cutoff score was adjusted to ≥18 for SYFPEITHI and <50 nM for EpiJen*.

### Construction and Characterization of Nanoformulations Based on PLGA NPs

Poly(lactic-*co*-glycolic) acid 75:25 (Resomer RG752H, MW: 4–15 kDa), polyvinyl alcohol (PVA; MW: 30–70 kDa, 87–90% hydrolyzed), MPLA from *Salmonella minnesota*, phosphate-buffered saline (PBS; 10×, pH 7.4), *N*-(3-Dimethylaminopropyl)-*N*′-ethylcarbodiimide hydrochloride (EDC), and *N*-hydroxysuccinimide 98% (NHS) were purchased from Sigma-Aldrich (Vienna, Austria). All the other reagents were of analytical grade and commercially available. PLGA NPs containing the chimeric peptide (chCPAp, chH1p, or chKMP-11p) and the adjuvant MPLA were prepared by the double emulsion method, as previously described (32). Briefly, 2.9 ml of a PLGA chloroform solution (31 mg/ml) were mixed with 0.1 ml of an MPLA solution (10 mg/ml) in methanol:chloroform (1:4 v/v). Water-in-oil (w/o) emulsion was then formed by adding 0.3 ml of the chimeric peptide solution in PBS at a final concentration of 6.6 mg/ml into the PLGA/MPLA solution. The emulsification was performed in an ice bath with the aid of a microtip sonicator (Vibra Cell VC-505; Sonics, Newtown, CO, USA) at 40% amplitude for 45 s. Subsequently, the primary emulsion (w/o) was added into 12 ml of 1% (w/v) PVA aqueous solution. The mixture was then emulsified *via* sonication at 40% amplitude for 2 min. The resulting double (w/o/w) emulsion was stirred overnight to allow the evaporation of chloroform. The PLGA NPs were then purified by means of four successive centrifugation–redispersion cycles in sterilized water, at 13,860 × *g* for 10 min at 4°C and were subsequently lyophilized (ScanVac Freezedryers CoolSafe 55-9; LaboGene ApS, Lynge, Denmark). For the preparation of the PLGA NPs loaded with each of the chimeric peptides, the same volume of peptide solution as previously was added into 3 ml of a PLGA chloroform solution at a final concentration of 30 mg/ml. Lyophilized PLGA NPs were stored at 4°C.

For specific purposes, PLGA NPs were surface modified with a synthetic octapeptide (p8: CYTYQGKL; JPT, Berlin, Germany) that mimics the TNFα-docking region with the TNFRII. The peptide was conjugated to NPs *via* a two-step carbodiimide method (33, 34). Accordingly, 1.5 ml of a 7 wt% EDC solution and 1.5 ml of a 0.3 wt% NHS solution both prepared in 20 mM HEPES/NaOH buffer containing 1% (v/v) Pluronic F-68 at pH 7 were added into 1 ml of PLGA NPs (empty or loaded with each of the chimeric peptides) dispersion in the same buffer at a final concentration of 20 mg/ml, in order to activate the PLGA carboxyl groups (35). The mixture was then stirred end-over-end for 2 h at room temperature. The residual reagents were removed by centrifugation at 13,860 × *g* for 10 min at 25°C. Subsequently, 0.3 ml of 0.1% (w/v) p8 solution in 20 mM HEPES/NaOH buffer containing 1% (v/v) Pluronic F-68 at pH 7 were added to the PLGA NPs dispersion, and the mixture was incubated for 18 h at room temperature. To saturate free-coupling sites, 500 µL of 20% (w/v) glycine in 20 mM HEPES/NaOH buffer containing 1% (v/v) Pluronic F-68 at pH 7.0 was added and incubated end-over-end for 1 h at 25°C. The PLGA NPs were subsequently purified by means of two successive centrifugation–redispersion cycles at 13,860 × *g* for 10 min at 25°C in the same buffer. The finally collected NPs were subsequently lyophilized and stored at 4°C.

The surface morphology of the PLGA NPs was observed by scanning electron microscopy (SEM) (JEOL JSM 6300). Accordingly, the lyophilized NPs were first double coated with a gold layer under vacuum and then examined by SEM. The average particle diameter of the PLGA NPs was determined by photon correlation spectroscopy and their zeta potential by aqueous electrophoresis measurements (Nano ZS90; Malvern Instruments Ltd., Malvern, UK). The measurements were performed with aqueous dispersions of NPs prior to their lyophilization.

### Quantification of Antigen, Adjuvant, and Targeting Ligand and *In Vitro* Release Studies

The MicroBCA Protein assay kit (Thermo Scientific, Rockford, IL, USA) was employed to determine the chimeric peptide load (wt%) in the PLGA NPs according to the manufacturer’s instructions. Briefly, 2.5 mg of lyophilized PLGA NPs was dissolved in 0.25 ml DMSO for 1 h following a further dissolution in 1.25 ml of 0.05 N NaOH/0.5% (v/v) SDS for 3 h at 25°C. PLGA NPs without encapsulated chimeric peptide were used as controls. The absorbance of the samples was measured at 562 nm using a microplate reader (EL808IU-PC, BioTek Instruments Inc., Winooski, VT, USA). Peptide encapsulation efficiency (%) was calculated by the ratio of the peptide mass in the PLGA NPs over the total mass of peptide used. Peptide load (wt%) was calculated by the ratio of the encapsulated mass of peptide over the total mass of PLGA NPs.

A Limulus Amebocyte Lysate (LAL) kit (Lonza, Walkersville, MD, USA) was used for the determination of the MPLA load (wt%) in the PLGA NPs according to the manufacturer’s instructions. Briefly, a standard curve was established using different concentrations of aqueous MPLA solutions ranging from 0.01 to 10 ng/ml, which was found to be linear for the MPLA concentration range used with a correlation coefficient of *R*^2^ = 0.9994. The encapsulation efficiency (%) of MPLA was calculated by the ratio of the measured MPLA mass in the PLGA NPs over the total mass of MPLA in the recipe. Similarly, the MPLA load (wt%) was calculated by the ratio of encapsulated MPLA mass over the total mass of the PLGA NPs.

A UV–Vis spectrophotometer (Lambda 35; PerkinElmer, Waltham, MA, USA) was used for the determination of p8 amount (wt%) conjugated on the PLGA NPs surface. Accordingly, 2.5 mg of lyophilized p8-conjugated PLGA NPs was dissolved in 0.25 ml DMSO for 1 h following a further dissolution in 1.25 ml 0.05 N NaOH/0.5% (v/v) SDS for 3 h. The absorbance of the samples was measured at 492 nm. The calibration curve was found to be linear over the p8 concentration range of 0.3125–70 µg/ml with a correlation coefficient of *R*^2^ = 0.9982. Conjugation efficiency (%) of p8 was calculated by the ratio of the measured p8 mass on the PLGA NPs over the total mass of p8 used. Similarly, p8 load (wt%) was calculated by the ratio of conjugated p8 mass over the total mass of the PLGA NPs.

For the assessment of the *in vitro* release of the chimeric peptides and MPLA, PLGA NPs were dispersed in PBS at a final concentration of 1 mg/ml and incubated at 37°C in a thermomixer (Eppendorf) under constant stirring at 120.7 × *g*. At predetermined time points (0, 1, 2, 4, 6, 12, 24, 48 h, and 1, 2 weeks), 1 ml of the dispersion was centrifuged at 13,860 × *g* for 10 min at 4°C. The supernatants were collected, and the amount of the chimeric peptide or MPLA was determined using the MicroBCA or the LAL kit, respectively.

### Experimental Animals and Ethics Statement

B6.Cg-Tg(HLA-A/H2-D)2Enge/J humanized transgenic mice, provided by the Jackson Laboratory (Bar Harbor, ME, USA), were used in this study. These transgenic mice express an interspecies hybrid class I MHC gene, *aad*, which contains the alpha-1 and alpha-2 domains of the human HLA-A2.1 gene and the alpha-3 transmembrane and cytoplasmic domains of the mouse H-2D^d^ gene, under the direction of the human HLA-A2.1 promoter, while they are created on a C57BL/6 background. BALB/c mice, also used in this study, were obtained from the breeding unit of the Hellenic Pasteur Institute (Athens, Greece). Both strains were reared in institutional facilities under specific pathogen-free conditions at ambient temperature of 25°C, receiving a diet of commercial food pellets and water *ad libitum*; female mice 6–8 weeks old were used. Animal protocols had been approved by the institutional Animal Bioethics Committee regulating according to the National Law 2013/56 and the EU Directive 2010/63/EU for animal experiments and complied with the ARRIVE guidelines. All efforts were made to minimize animal suffering.

### Generation and Characterization of DCs

Dendritic cells were generated from pluripotent bone marrow stem cells obtained from HLA A2.1 transgenic mice in the presence of recombinant mouse granulocyte macrophage colony-stimulating factor (rmGM-CSF) as previously described (36). Briefly, 3.5 × 10^6^ bone marrow cells obtained from the tibias and femurs of mice were seeded in a 100 mm Petri dish in 10 ml of RPMI-1640 medium (Biochrom AG, Berlin, Germany) supplemented with 2 mM l-glutamine, 10 mM HEPES, 100 U/ml penicillin, 100 µg/ml streptomycin (complete RPMI-1640 medium), 10% (v/v) heat-inactivated fetal bovine serum (FBS; Gibco, Paisley, UK), and 20 ng/ml rmGM-CSF (Peprotech, London, UK) and cultured for 7 days at 37°C and 5% CO_2_. On days 3 and 6, loosely adherent cells were harvested and resuspended in fresh culture medium supplemented with the same dose of rmGM-CSF. On day 7, non-adherent cells were collected and characterized; cell viability was >95% as determined by trypan blue exclusion, and the percentage of CD11c^+^CD8α^−^ cells was >85% as assessed by R-phycoerythrin (R-PE)-conjugated hamster anti-mouse CD11c (clone HL3; BD Biosciences, Erembodegem, Belgium) and FITC-conjugated rat anti-mouse CD8α (clone Lyt2; BD Biosciences) monoclonal antibodies (mAbs) and flow cytometry. For each sample, 10,000 cells were analyzed on a FACS Calibur (Becton-Dickinson, San Jose, CA, USA), and the data were processed using FlowJo V10 software (Tree Star Inc., Ashland, OR, USA).

### Stimulation of DCs and Analysis of Their Maturation and Functional Differentiation Profile by Flow Cytometry

To analyze the effect of differentially functionalized PLGA nanoformulations on DCs maturation and functional differentiation, DCs were harvested on day 7, seeded in 24-well plates at a density of 1 × 10^6^ cells/ml, and cultured for 24 h at 37°C and 5% CO_2_ in the presence of (i) PLGA, (ii) PLGA-MPLA, (iii) p8-PLGA, (iv) PLGA-chCPAp, (v) PLGA-chH1p, (vi) PLGA-chKMP-11p, (vii) a mix of PLGA-chCPAp, PLGA-chH1p, and PLGA-chKMP-11p (mix A), (viii) PLGA-chCPAp-MPLA, (ix) PLGA-chH1p-MPLA, (x) PLGA-chKMP-11p-MPLA, (xi) a mix of PLGA-chCPAp-MPLA, PLGA-chH1p-MPLA, and PLGA-chKMP-11p-MPLA (mix B), (xii) p8-PLGA-chCPAp, (xiii) p8-PLGA-chH1p, (xiv) p8-PLGA-chKMP-11p, and (xv) a mix of p8-PLGA-chCPAp, p8-PLGA-chH1p, and p8-PLGA-chKMP-11p (mix C). In parallel, DCs were cultured under the same conditions in the presence of each soluble chimeric peptide or a mix of soluble chimeric peptides with or without the adjuvant MPLA (mix D or mix E, respectively). Unstimulated DCs or DCs stimulated with 1 µg/ml LPS (Sigma-Aldrich) were used as negative and positive control, respectively. The optimal dose for each chimeric peptide encapsulated in PLGA NPs was determined at 2 µg according to preliminary experiments (data not shown). At the end of incubation, cells were washed with FACS buffer [3% (v/v) FBS in PBS] and then labeled with R-PE-conjugated 1:100 diluted rat anti-mouse CD40 (clone 3/23; BD Biosciences), hamster anti-mouse CD80 (clone 16-10A1; BD Biosciences), rat anti-mouse CD86 (clone GL-1; BD Biosciences), mouse anti-mouse MHCI H-2 Kb/H-2 Db (clone 5041.16.1; Acris, Herford, Germany), or 1:200 diluted rat anti-mouse MHCII I-Ab chain, H2-Eb1 (clone NIMR; Acris), or 1:10 diluted mouse anti-human HLA-ABC (clone G46-2.6; BD Biosciences) mAbs for 30 min at 4°C in the dark. For intracellular staining, cells were subjected to 2.5 µg/ml brefeldin A (Applichem, Darmstadt, Germany) during the last 4 h of culture and then they were fixed with 2% (w/v) paraformaldeyde (PFA; Sigma-Aldrich) in PBS and stained with PE-conjugated 1:100 diluted anti-mouse IL-12p40/p70 (clone C15.6; BD Biosciences) mAb in permeabilization buffer [0.1% (v/v) saponin in FACS buffer] for 30 min at 4°C in the dark. After a washing step with FACS buffer, cells were analyzed by flow cytometry, and data were processed as previously described.

### Mixed Leukocyte Reaction (MLR) Induced by Stimulated DCs

Spleens from BALB/c mice were aseptically excised and teased into single-cell suspension in complete RPMI-1640 medium supplemented with 10% (v/v) FBS. Red blood cells were removed by treating spleen cells with ammonium chloride lysis solution, pH 7.2 (ACK; 0.15 M NH_4_Cl, 1 mM KHCO_3_, 0.1 mM Na_2_EDTA). Lysis reaction was stopped by adding ice-cold complete RPMI-1640 medium, and spleen cells were washed twice. DCs that have been stimulated as described above for 24 h were washed and co-cultured with 2 × 10^5^ naive spleen cells at different ratios (1:5, 1:10, or 1:20) in 96-well round-bottom plates for 96 h at 37°C and 5% CO_2_. Spleen cells cultured in medium alone or in the presence of 6 µg/ml concanavalin A (Con A; Sigma-Aldrich) served as negative and positive control of T cell proliferation, respectively. Cells were pulsed with 1 μCi/ml of ^3^[H]-thymidine (^3^[H]-TdR; PerkinElmer, MA, USA) for the last 18 h of the culture period and then were harvested. The ^3^[H]-TdR incorporation was determined on a microplate scintillation counter (Microbeta Trilux, Wallac, Turku, Finland). All samples were run in triplicate.

In parallel, DCs stimulated for 24 h with (i) the mix of PLGA-chCPAp, PLGA-chH1p, and PLGA-chKMP-11p nanoformulations (mix A), (ii) the mix of PLGA-chCPAp-MPLA, PLGA-chH1p-MPLA, and PLGA-chKMP-11p-MPLA nanoformulations (mix B), (iii) the mix of p8-PLGA-chCPAp, p8-PLGA-chH1p, and p8-PLGA-chKMP-11p nanoformulations (mix C), (iv) the mix of soluble chimeric peptides (mix D), and (v) the mix of the soluble chimeric peptides with the adjuvant MPLA (mix E) were co-cultured with 1 × 10^6^ naïve spleen cells from BALB/c mice at a ratio of 1:5 in 24-well plates for 48 h at 37°C and 5% CO_2_. Cells were exposed to 2.5 µg/ml brefeldin A during the last 4 h of culture and then washed with FACS buffer and fixed with 2% (w/v) PFA in PBS. Fixed cells were stained with APC-conjugated hamster anti-mouse CD3e (clone 145-2C11), FITC-conjugated rat anti-mouse CD4 (clone RM4-5) or CD8α (clone 53-6.7) mAbs, and R-PE-conjugated rat anti-mouse IFNγ (clone XMG1.2) or IL-4 (clone BVD4-1D11) mAbs in permeabilization buffer for 30 min at 4°C in the dark. All mAbs used in the protocol were purchased from BD Biosciences. After a washing step with FACS buffer, 20,000 cells from each sample were analyzed by flow cytometry, and data were processed as previously described.

### RNA Extraction and Microarray Assay

To analyze the effect of differentially functionalized PLGA nanoformulations on DCs gene expression, DCs were stimulated with (i) the mix of PLGA-chCPAp, PLGA-chH1p, and PLGA-chKMP-11p nanoformulations (mix A), (ii) the mix of PLGA-chCPAp-MPLA, PLGA-chH1p-MPLA, and PLGA-chKMP-11p-MPLA nanoformulations (mix B), (iii) the mix of p8-PLGA-chCPAp, p8-PLGA-chH1p, and p8-PLGA-chKMP-11p nanoformulations (mix C), and (iv) the mix of soluble chimeric peptides (mix D) at 37°C and 5% CO_2_, for 18 h. DCs cultured in medium alone were used as a reference group. Total RNA was isolated with Trizol Reagent (Invitrogen, Karlruhe, Germany) and purified on a Qiagen RNeasy column (Qiagen, Hilden, Germany) according to the manufacturer’s instructions. RNA was quantified with ND-1000 Nanodrop (Thermo Fisher Scientific, Wilmington, DE, USA), and RNA integrity was assessed using the RNA 6000 NanoLabChip kit on Agilent Bioanalyzer 2100 (Agilent Technologies, Inc., Palo Alto, CA, USA). Only samples with intact total RNA profiles (retention of both ribosomal bands and the broad central peak of mRNA) and RIN > 7.0 were utilized in microarray analysis. Approximately 300 ng of total RNA were used to generate biotinylated complementary RNA (cRNA) for each group using the GeneChip^®^ WT PLUS Reagent Kit protocol for whole transcript (WT) expression array, Rev3. Poly-A RNA control added to the RNA test samples as exogenous positive control, and the RNA was reverse transcribed to double-stranded cDNA, and biotinylated cRNA was synthesized and purified according to the protocol. A second cycle of cDNA synthesis followed along with a second purification step. Then, 6 µg of the single-stranded DNA was fragmented, labeled with the appropriate labeling reagent, and hybridized to GeneChip^®^ Mouse Gene 2.0 ST arrays (Affymetrix, UK). Hybridization took place for 16 h in an Affymetrix GeneChip^®^ Hybridization Oven 640. Affymetrix GeneChip^®^ Fluidics Station 450 was used to wash and stain the arrays with streptavidin–phycoerythrin according to the standard antibody amplification protocol for eukaryotic targets. Arrays were scanned on Affymetrix GeneChip^®^ Scanner 3000 at 570 nm. The Affymetrix eukaryotic hybridization control and Poly-A RNA control were used to ensure efficiency of hybridization and cRNA amplification. Images and data were acquired and analyzed using the Affymetrix^®^ GeneChip^®^ Command Console^®^ Software (AGCC), where initial quality control of the experiment was performed. AGCC was also used to perform robust multi-array average (RMA) normalization and probe set summarization steps on the raw signal intensity files. Principal component analysis (PCA) and heatmap representation of individual values were conducted utilizing appropriate open-source software. Differential expression analysis was performed for stimulated vs. unstimulated DCs utilizing one-way ANOVA (*p* < 0.05) and a ±0.585log2 (fold change) threshold on mean gene-expression levels. Probe sets that were unannotated or that mapped to the same gene in a many-to-one fashion were removed from the subsequent analysis. Microarray raw files and RMA-summarized data have been deposited in Gene Expression Omnibus under accession number GSE92869.

### Functional Analysis of Gene-Expression Profiling

Mapping of array-specific probe sets to Entrez Gene IDs was conducted using up-to-date annotation package mogene20sttranscriptcluster.db in R. Significantly deregulated genes [*p* < 0.05, ±0.585log2 (fold change) threshold] from each comparison (mix A–D vs. reference) were subjected to functional enrichment analysis with the R package *clusterProfiler* (37), setting parameters “pvalueCutoff” = 0.05 and “pAdjustMethod” = “fdr” for multiple comparisons. Terms from Gene Ontology (Biological Process) database slice (38) were tested for enrichment. Mappings between GO terms and Entrez Gene IDs relied on regularly updated R package org.Mm.eg.db. R package *Pathview* (39) was utilized to superimpose differentially expressed genes upon KEGG pathways. Red and green colors signify upregulation and downregulation on stimulated DCs, respectively.

### Immunization and Analysis of Antigen-Specific T Cell Responses

HLA A2.1 transgenic mice (*n* = 5) were immunized subcutaneously in the upper and dorsal region with the mix of PLGA-chCPAp-MPLA, PLGA-chH1p-MPLA, and PLGA-chKMP-11p-MPLA nanoformulations (mix B) in a total volume of 100 µl sterile PBS. Taking into account the chimeric peptide and MPLA loads of the above nanoformulations, each mouse received in total 6 µg of chimeric peptides (2 µg of each chimeric peptide) with 3 µg MPLA. Two booster doses—also subcutaneously injected—followed at a 2-week interval. Control groups (*n* = 5 mice/group) received PLGA-MPLA nanoformulations in PBS or only PBS.

Two weeks after the last immunization, mice were euthanized, and spleens were removed aseptically. To analyze antigen-specific T cell proliferation, single-cell suspensions were prepared in complete RPMI-1640 medium supplemented with 10% (v/v) FBS, as previously described. Spleen cells were cultured in 96-well round bottom plates at a density of 2 × 10^5^ cells/200 μl/well in the presence of 5 µg/ml soluble chCPAp or chH1p or chKMP-11p for 96 h at 37°C and 5% CO_2_. Spleen cells cultured in medium alone or in the presence of 6 µg/ml Con A served as negative and positive control of T cell proliferation, respectively. Cells were pulsed with 1 μCi/ml of ^3^[H]-TdR for the last 18 h of the culture period and then were harvested. The ^3^[H]-TdR incorporation was determined on a microplate scintillation counter. All samples were run in triplicate, and the results were expressed as Δcpm (cpm of stimulated spleen cells − cpm of unstimulated spleen cells).

In parallel, intracellular cytokine staining and flow cytometry were performed in the spleen cell suspensions from immunized mice in order to identify antigen-specific IFNγ-producing CD8^+^ T cells. In brief, 1 × 10^6^ spleen cells were cultured in 24-well plates in the presence of 5 µg/ml soluble chCPAp or chH1p or chKMP-11p for 48 h, according to previously described protocols (29, 40), at 37°C and 5% CO_2_. Spleen cells cultured in medium alone served as negative control. Spleen cells were exposed to 2.5 µg/ml brefeldin A during the last 4 h of culture and then washed with FACS buffer and fixed with 2% (w/v) PFA in PBS. After fixation, spleen cells were stained with APC-conjugated anti-CD3, PE-conjugated anti-IFNγ, and FITC-conjugated anti-CD8 mAbs in permeabilization buffer and analyzed with flow cytometry, as previously described.

### Parasites, Infection Protocol, and Evaluation of Parasite Burden by a Limiting Dilution Assay

A strain of *L. infantum* (MHOM/GR/2001/GH8) originally isolated from a Greek patient suffering from VL (41) was cultured *in vitro* and maintained infective through serial passage in BALB/c mice, as previously described (36). Immunized with the mix B and non-immunized (received only PBS) HLA A2.1 transgenic mice (*n* = 5/group) were infected by injecting intravenously 2 × 10^7^ stationary phase *L. infantum* promastigotes in 100 µl PBS, 2 weeks after the final booster dose. Mice immunized with PLGA-MPLA nanoformulations and then infected were used as a control group. The protective effect of immunization with the mix B was assessed in liver and spleen of the infected mice at 1 and 2 months post-infection. The protective effect was determined in comparison with the non-immunized control group according to the formula: percentage of reduction in parasite burden = (number of parasites from the non-immunized infected control group − number of parasites from the immunized infected group)/(number of parasites from the non-immunized infected control group) × 100.

The parasite burden was assessed by a limiting dilution assay following well-established protocols (42) with minor modifications. Briefly, livers and spleens were removed aseptically from the euthanized mice and weighed. Suspensions of liver or spleen tissue were prepared in Schneider’s *Drosophila* medium (Biosera, Boussens, France) supplemented with 100 U/ml penicillin, 100 µg/ml streptomycin, and 20% (v/v) FBS to 1 mg/ml final concentration. Serial twofold dilutions of the tissue suspensions were plated in 96-well culture plates and incubated at 26°C for 7 days. After the incubation period, the presence or absence of viable and motile promastigotes was recorded in each well. The reciprocal of the highest dilution that was positive for parasites was considered to be the number of parasites per milligram of tissue. The total parasite burden was calculated by reference to the whole organ weight.

### Statistical Analysis

Results are derived from at least two independent experiments and are expressed as the mean value ± SD. One-way ANOVA and Tukey’s multiple comparisons test or two-way ANOVA and Bonferroni multiple comparisons test were performed, when required, to assess statistical differences using the GraphPad Prism software (version 5.0; San Diego, CA, USA). The probability (*p*) of <0.05 was considered to indicate statistical significance.

## Results

### Design and Construction of Experimental Peptide-Based Nanovaccines

#### Design of Chimeric Peptides from the Immunogenic *L. infantum* Proteins CPA, Histone H1, and KMP-11

In a previous study based on an *in silico* analysis of the *L. infantum* proteins CPA, histone H1, and KMP-11, multi-epitope peptides were designed in a way that each peptide contained at least one HLA class I-restricted epitope scored very high, as well as adjacent or overlapping HLA class II-restricted epitopes scored also high (29). All the multi-epitope peptides also contained more than one epitopes with high-binding affinity to HLA A*0201 (Table 2), the most prevalent among HLA A2 allelic variants (43) with 72.2% coverage in Caucasians (44). The multi-epitope peptide sequences were also checked for the presence of H2-Db- or H2-Kb-restricted epitopes. No epitopes were predicted with binding affinity to H2-Kb allele, whereas a small number of epitopes were predicted with binding affinity to H2-Db allele with great overlap with the HLA A*0201 epitopes. Selected multi-epitope peptides were linked together with proper aa linkers in order to design longer chimeric peptides (Table 1). More specifically, the tripeptide AAY was used as a linker between the multi-epitope peptides of CPA (CPA_p2 and CPA_p3) and histone H1 (H1_p1 and H1_p3), while the motif HEYGAEALERAG was used as a linker between the multi-epitope peptide of KMP-11 (KMP-11_p1) and PADRE, a synthetic pan HLA DR-binding epitope of 13 residues (AKFVAAWTLKAAA) that binds to a wide spectrum of human and mouse MHC class II alleles and induce T_H_ cell responses.

#### Physicochemical Characterization of PLGA-Based Antigen-Delivery Systems

Chimeric peptides were synthesized and each of them was encapsulated in PLGA NPs alone or in combination with the MPLA adjuvant, or in PLGA NPs surface modified with the octapeptide p8. The different nanoformulations and their characteristics are summarized in Table 3. Both chimeric peptide-loaded and chimeric peptide/MPLA-loaded PLGA NPs had an average diameter in the range of 291.4–413.5 nm and a negative zeta potential value, varying from −31.6 to −10.6 mV. The negative zeta potential values of the blank PLGA NPs are due to the carboxyl groups residing on the surface of NPs that are negatively charged at physiological pH (45). The observed slight decrease in the absolute zeta potential value of the chimeric peptide loaded PLGA NPs can be attributed to the presence of antigen that partially neutralizes the free anionic surface carboxyl groups. On the other hand, the negative zeta potential values observed for the chimeric peptide/MPLA-loaded PLGA NPs are due to the combined negative charges of the PLGA carboxyl groups and MPLA molecules that have a zeta potential value of approximately −50 mV in water. Further, PLGA was successfully conjugated with p8 resulting in partial neutralization of the free anionic carboxyl groups residing on the PLGA NPs surface and thus explaining the decrease of the absolute values of zeta potential of the functionalized NPs (−4.0 to −5.5 mV) (46).

**Table 3:** Properties of synthesized nanoformulations based on poly(lactic-*co*-glycolic) acid (PLGA) nanoparticles.

Formulation name	Average size (nm)	*z* potential (mV)	Peptide load (wt%)	Peptide EE (%)	Monophosphoryl lipid A (MPLA) load (wt%)	MPLA EE (%)	p8 load (wt%)	p8 CE (%)
PLGA-chCPAp	291.4 ± 3.1	−31.6 ± 2.0	1.84 ± 0.04	86.44 ± 1.86	–	–	–	–
PLGA-chH1p	341.8 ± 2.8	−15.0 ± 0.2	1.41 ± 0.20	64.59 ± 7.10	–	–	–	–
PLGA-chKMP-11p	341.0 ± 1.2	−20.1 ± 0.2	1.84 ± 0.10	84.57 ± 2.37	–	–	–	–
PLGA-chCPAp-MPLA	374.5 ± 12.5	−16.1 ± 0.2	1.89 ± 0.03	87.35 ± 1.35	0.50 ± 0.06	45.44 ± 5.02	–	–
PLGA-chH1p-MPLA	413.5 ± 4.5	−10.6 ± 0.1	1.44 ± 0.08	65.30 ± 2.04	0.98 ± 0.01	92.85 ± 1.05	–	–
PLGA-chKMP-11p-MPLA	299.9 ± 1.05	−19.8 ± 0.1	1.80 ± 0.06	82.06 ± 0.99	0.81 ± 0.06	75.19 ± 4.80	–	–
p8-PLGA-chCPAp	392.2 ± 0.8	−4.0 ± 0.1	1.84 ± 0.04	86.44 ± 1.86	–	–	0.83 ± 0.14	49.62 ± 5.41
p8-PLGA-chH1p	421.1 ± 2.1	−5.5 ± 0.1	1.41 ± 0.20	64.59 ± 7.10	–	–	0.77 ± 0.09	53.06 ± 3.15
p8-PLGA-chKMP-11p	424.1 ± 3.8	−4.3 ± 0.2	1.84 ± 0.10	84.57 ± 2.37	–	–	0.73 ± 0.08	51.68 ± 2.90

*Results are presented as mean ± SD (*n* = 3)*.

*EE, encapsulation efficiency; CE, conjugation efficiency*.

**Figure 1:**
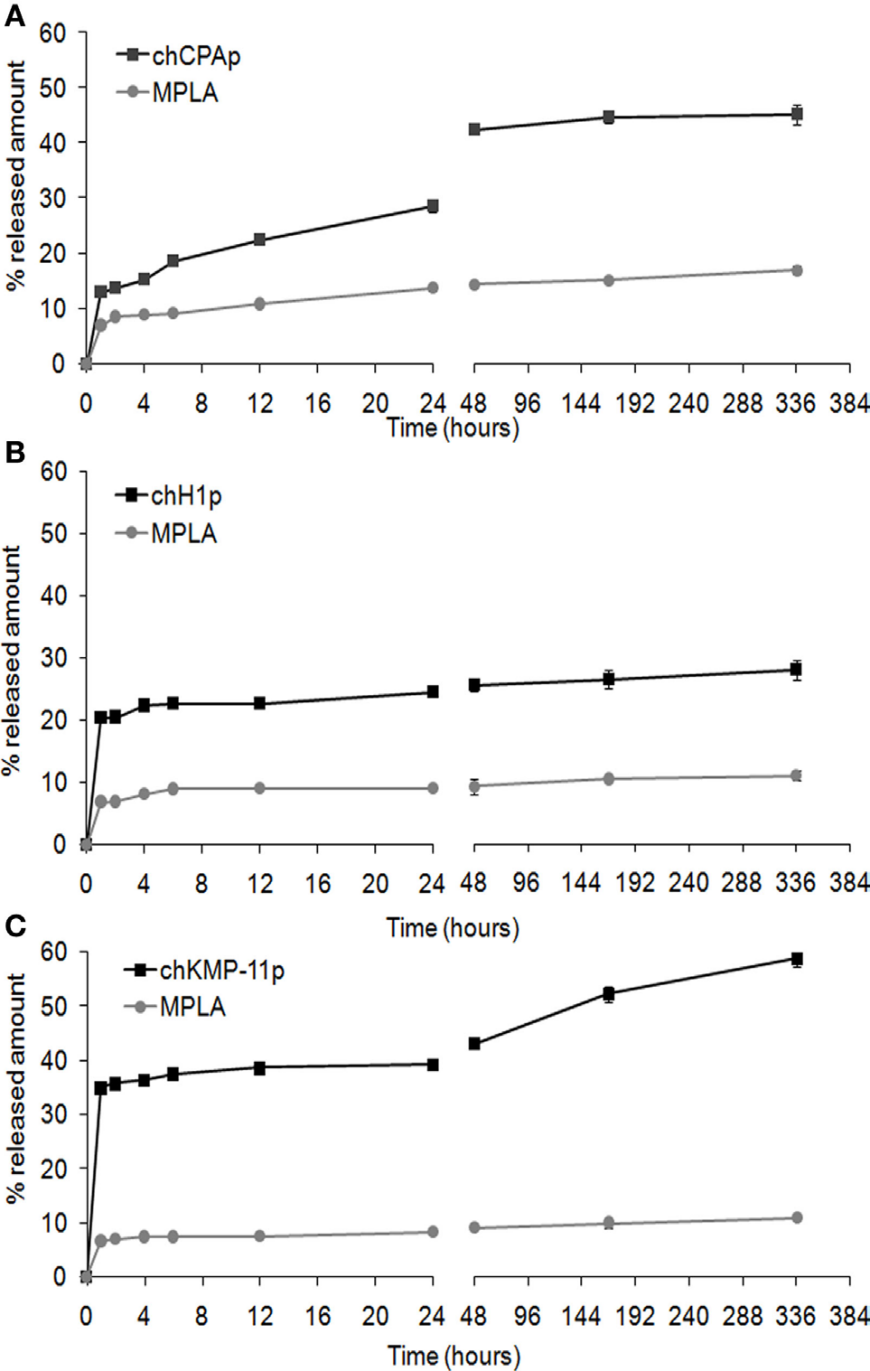
*In vitro* release profile of the chimeric peptides from peptide-based poly(lactic-*co*-glycolic) acid (PLGA) nanoformulations. Time-dependent release of chCPAp and monophosphoryl lipid A (MPLA) adjuvant from PLGA-chCPAp-MPLA nanoformulations **(A)**, chH1p and MPLA adjuvant from PLGA-chH1p-MPLA nanoformulations **(B)**, and chKMP-11p and MPLA adjuvant from PLGA-chKMP-11p-MPLA nanoformulations **(C)** into PBS at 37°C. Data represent the mean ± SD of three independent experiments.

The *in vitro* release profiles of each chimeric peptide and MPLA from the PLGA NPs in PBS at 37°C are shown in Figure 1. About 45% of the total amount of chCPAp (Figure 1A) and chKMP-11p (Figure 1C) and 25% of the total amount of chH1p (Figure 1B) are released from the PLGA NPs during the first hour, possibly reflecting the release of antigen located near the NPs external surface and/or in the pores connected to the surface. This initial burst release of antigen was followed by a phase of slight sustained release. Thus, approximately 45% of chCPAp, 30% of chH1p, and 60% of chKMP-11p were released after 2 weeks since the polymer matrix was not completely hydrolyzed during this period. Regarding MPLA, it is apparent that it exhibits a similar release profile to that of the chimeric peptides. However, only 10–20% of the total MPLA amount was released after 2 weeks of incubation in PBS at 37°C.

### The MPLA Incorporation in PLGA Nanoformulations or the Surface Modification with p8 Induced a Strong DCs Maturation Profile

**Figure 2:**
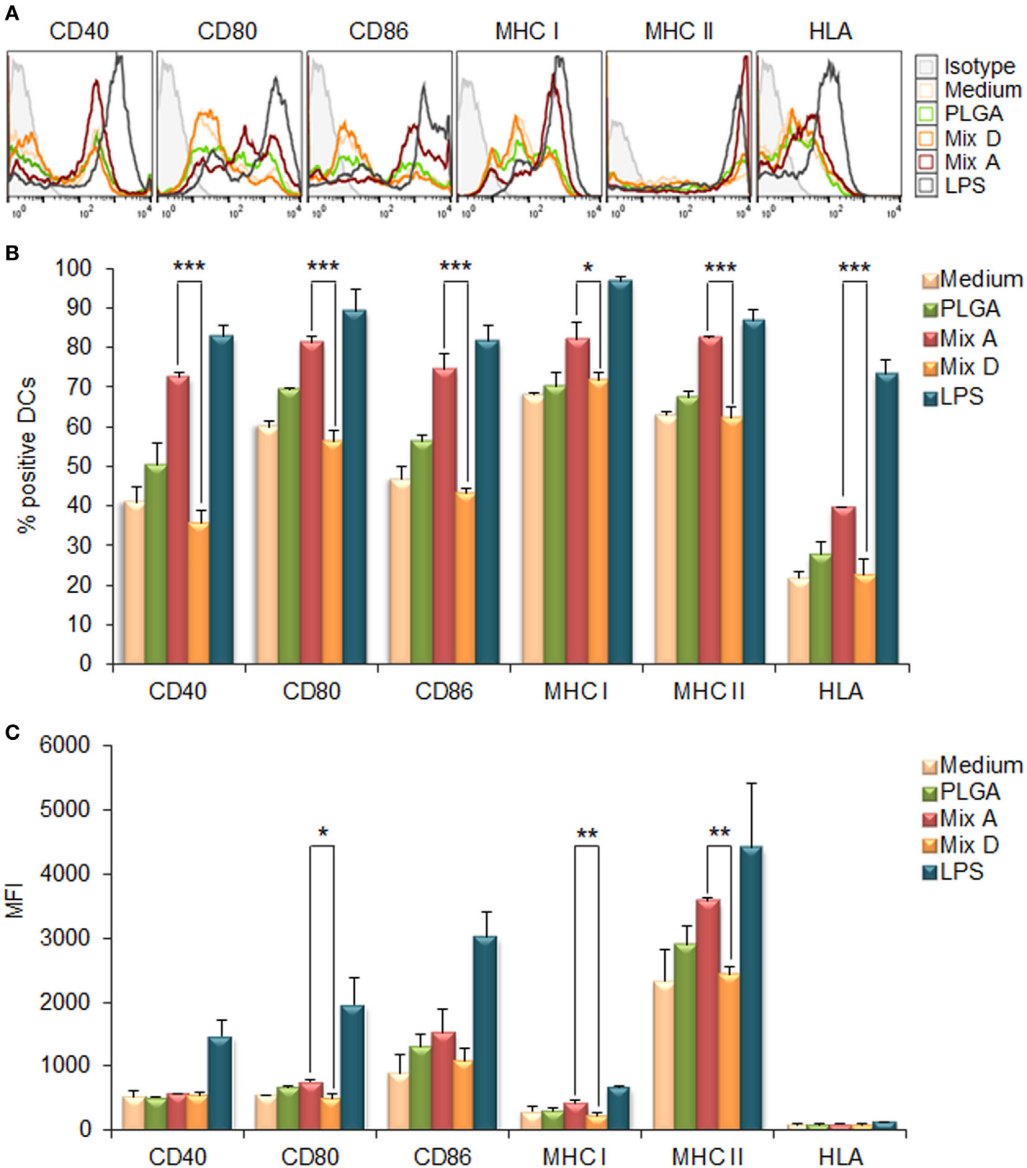
Encapsulation of chimeric peptides in poly(lactic-*co*-glycolic) acid (PLGA) nanoparticles (NPs) induced a strong maturation profile in dendritic cells (DCs) from HLA A2.1 transgenic mice. DCs were cultured for 24 h upon stimulation with the mix of PLGA-chCPAp, PLGA-chH1p, and PLGA-KMP-11p nanoformulations (mix A), the mix of soluble chimeric peptides (mix D), or empty PLGA NPs (control group). DCs cultured in medium alone or in the presence of 1 µg/ml LPS were used as negative and positive control, respectively. **(A)** Representative histogram plots of CD40, CD80, CD86, major histocompatibility complex (MHC) class I, and class II molecules levels. **(B)** Diagram demonstrating the percentage (%) of DCs expressing the co-stimulatory and MHC class I and class II molecules. **(C)** Diagram demonstrating the mean fluorescence index (MFI) of DCs expressing the co-stimulatory and MHC class I and class II molecules. Results are expressed as the mean ± SD of three independent experiments. Significant differences are indicated by *p < 0.05, **p < 0.01, or ***p < 0.001.

The capacity of the synthesized PLGA-based nanoformulations to directly activate and induce maturation of CD11c^+^ bone marrow-derived DCs after 24 h stimulation was assessed by flow cytometry, measuring the surface expression of the hallmark DCs maturation and T-cell co-stimulation markers MHC class I, MHC class II, CD40, CD80, and CD86. According to preliminary experiments, the optimum dose of each chimeric peptide encapsulated in PLGA NPs for efficient DCs maturation was determined at 2 µg (data not shown). As depicted in Figures 2A,B, the encapsulation of chimeric peptides in PLGA NPs resulted in a significant increase in the number of DCs expressing CD40, CD80, CD86, MHC class I, and MHC class II molecules, in comparison to DCs stimulated with soluble peptides (CD40: 72.40 ± 1.27 vs. 35.53 ± 3.41%, *p* < 0.001; CD80: 81.20 ± 1.56 vs. 56.20 ± 2.78%, *p* < 0.001; CD86: 74.33 ± 3.99 vs. 43.20 ± 1.13%, *p* < 0.001; murine MHC class I: 82.15 ± 4.31 vs. 71.60 ± 2.26%, *p* < 0.05; murine MHC class II: 82.35 ± 0.35 vs. 62.03 ± 2.97%, *p* < 0.001; hybrid HLA class I: 39.50 ± 0.30 vs. 22.55 ± 3.82%, *p* < 0.001). Regarding the mean fluorescence index (MFI) values (Figure 2C), significant increase was observed only for CD80, murine MHC class I, and MHC class II molecules in comparison to DCs stimulated with soluble peptides (CD80: 754 ± 26.87 vs. 503 ± 70.29, *p* < 0.05; murine MHC class I: 432 ± 50.91 vs. 237 ± 27.58, *p* < 0.01; MHC class II: 3,990 ± 718.74 vs. 2,370 ± 15.56, *p* < 0.01). It must be noted that DCs stimulated with each of the PLGA-chCPAp, PLGA-chH1p, and PLGA-chKMP-11p nanoformulations exhibited a similar maturation profile to that of the DCs stimulated with the mix of these nanoformulations (mix A), in comparison to DCs stimulated with the relevant soluble chimeric peptide (Figure S1 in Supplementary Material). The above results confirm that PLGA NPs are promising delivery systems of immunogenic peptides in the development of peptide-based vaccines.

**Figure 3:**
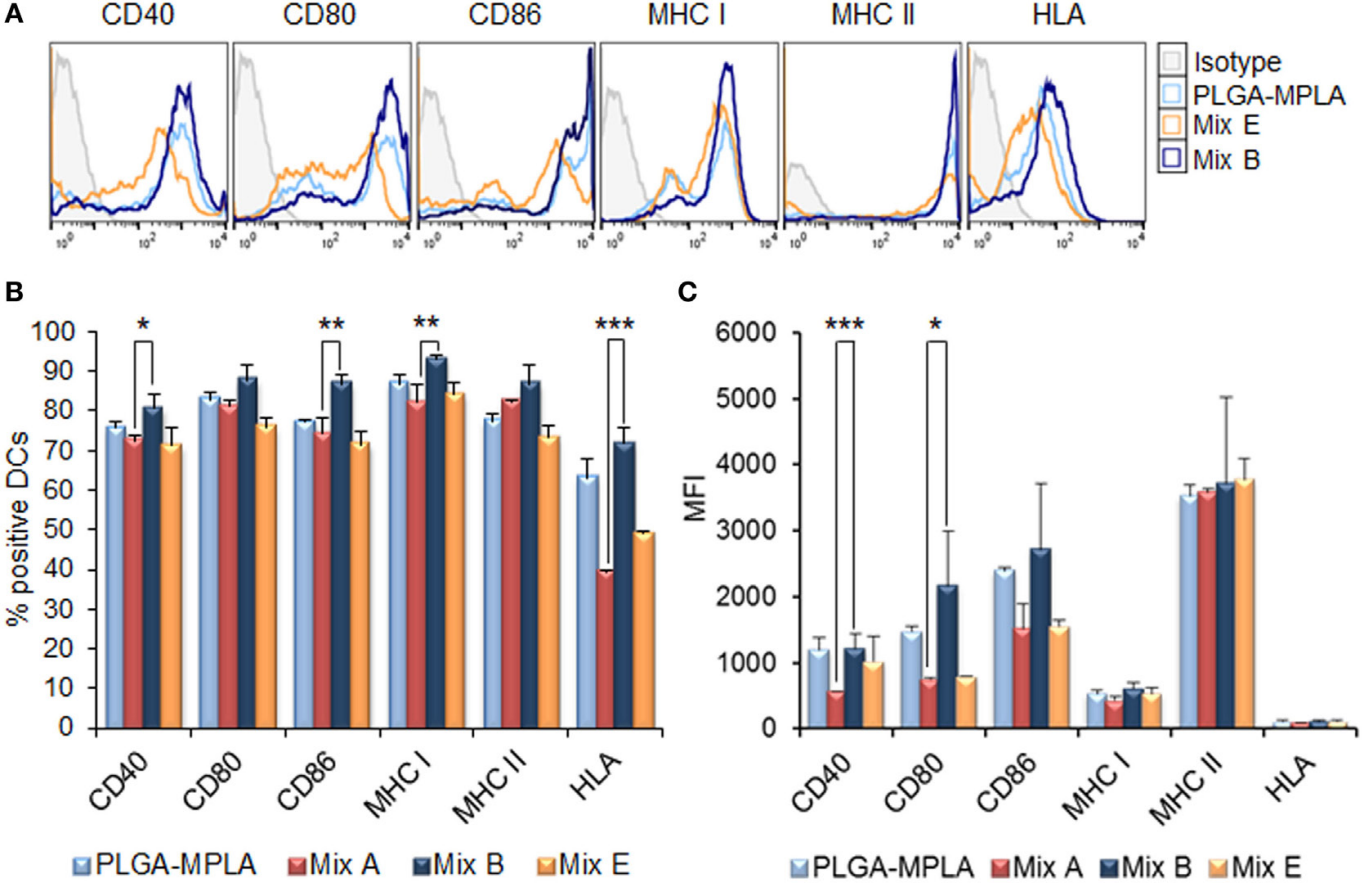
Monophosphoryl lipid A (MPLA) incorporation in peptide-based poly(lactic-*co*-glycolic) acid (PLGA) nanoformulations further enhanced the maturation of dendritic cells (DCs) from HLA A2.1 transgenic mice. DCs were cultured for 24 h upon stimulation with the mix of PLGA-chCPAp-MPLA, PLGA-chH1p-MPLA, and PLGA-KMP-11p-MPLA nanoformulations (mix B), the mix of soluble chimeric peptides with MPLA adjuvant (mix E), or PLGA nanoparticles with MPLA encapsulated. **(A)** Representative histogram plots of CD40, CD80, CD86, major histocompatibility complex (MHC) class I, and class II molecules levels. **(B)** Diagram demonstrating the percentage (%) of DCs expressing the co-stimulatory and MHC class I and class II molecules. **(C)** Diagram demonstrating the mean fluorescence index (MFI) of DCs expressing the co-stimulatory and MHC class I and class II molecules. Results are expressed as the mean ± SD of three independent experiments. Significant differences are indicated by *p < 0.05, **p < 0.01, or ***p < 0.001.

**Figure 4:**
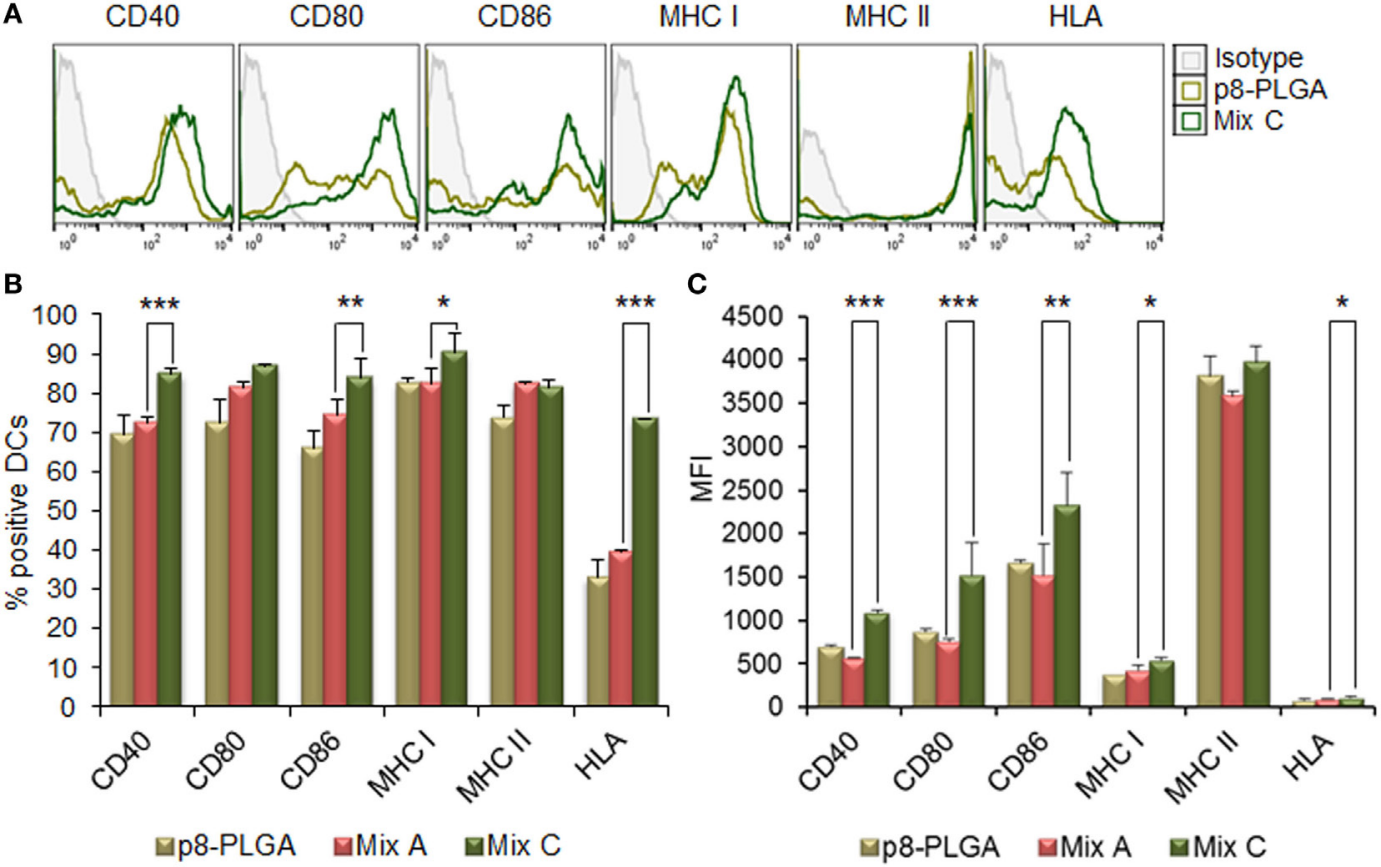
Surface modification of peptide-based poly(lactic-*co*-glycolic) acid (PLGA) nanoformulations with p8 also enhanced the maturation of dendritic cells (DCs) from HLA A2.1 transgenic mice. DCs were cultured for 24 h upon stimulation with the mix of p8-PLGA-chCPAp, p8-PLGA-chH1p, and p8-PLGA-KMP-11p nanoformulations (mix C) or PLGA nanoparticles surface modified with p8. **(A)** Representative histogram plots of CD40, CD80, CD86, major histocompatibility complex (MHC) class I, and class II molecules levels. **(B)** Diagram demonstrating the percentage (%) of DCs expressing the co-stimulatory and MHC class I and class II molecules. **(C)** Diagram demonstrating the mean fluorescence index (MFI) of DCs expressing the co-stimulatory and MHC class I and class II molecules. Results are expressed as the mean ± SD of three independent experiments. Significant differences are indicated by *p < 0.05, **p < 0.01, or ***p < 0.001.

The MPLA incorporation as well as the surface modification of PLGA NPs with p8 further increased DCs maturation. More specifically, as shown in Figures 3A,B, stimulation of DCs with the mix of PLGA-chCPAp-MPLA, PLGA-chH1p-MPLA, and PLGA-chKMP-11p-MPLA nanoformulations (mix B) led to a significant increase in the number of DCs expressing the co-stimulatory molecules CD40 (80.73 ± 3.46 vs. 72.40 ± 1.27%, *p* < 0.05) and CD86 (86.75 ± 2.33 vs. 74.33 ± 3.99%, *p* < 0.01), as well as the MHC class I molecules (murine MHC class I: 93.05 ± 0.78 vs. 82.15 ± 4.31%, *p* < 0.01; hybrid HLA class I: 71.65 ± 4.17 vs. 39.50 ± 0.30%, *p* < 0.001), in comparison to DCs stimulated with the mix A. This was also accompanied by a significant increase in the MFI values for CD40 (1,338 ± 81.32 vs. 618 ± 72.75, *p* < 0.0001) and CD80 (1,338 ± 81.32 vs. 618 ± 72.75, *p* < 0.0001) molecules (Figure 3C). Regarding the surface modification with p8, as depicted in Figures 4A,B, stimulation of DCs with the mix of p8-PLGA-chCPAp, p8-PLGA-chH1p, and p8-PLGA-chKMP-11p nanoformulations (mix C) significantly also enhanced the presence of DCs expressing the co-stimulatory molecules CD40 (84.80 ± 1.57 vs. 72.40 ± 1.27%, *p* < 0.001) and CD86 (83.60 ± 4.95 vs. 74.33 ± 3.99%, *p* < 0.01), as well as the MHC class I molecules (murine MHC class I: 90.25 ± 5.02 vs. 82.15 ± 4.31%, *p* < 0.05; hybrid HLA class I: 73.50 ± 0.05 vs. 39.50 ± 0.30%, *p* < 0.001), in comparison to DCs stimulated with the mix A. In addition, a significant increase was observed in the MFI values of DCs stimulated with the mix C in comparison to DCs stimulated with the mix A for all the co-stimulatory and MHC class I molecules except from the MHC class II molecule (CD40: 1,084 ± 28.93 vs. 618 ± 72.75, *p* < 0.0001; CD80: 1,715 ± 223.45 vs. 754 ± 26.87, *p* < 0.001; CD86: 2,529 ± 201.52 vs. 1,680 ± 347.19, *p* < 0.01; murine MHC class I: 537 ± 31.82 vs. 432 ± 50.91, *p* < 0.05; HLA class I: 122 ± 4.24 vs. 96 ± 2.26, *p* < 0.05). The above results concerning DCs stimulated with the mix B or the mix C were comparable to that observed in the case of DCs cultured in the presence of LPS (positive control of maturation; CD40: 82.80 ± 2.86%; CD80: 89.10 ± 5.61%; CD86: 81.63 ± 3.84%; murine MHC class I: 96.53 ± 1.34%; murine MHC class II: 86.63 ± 2.73%; hybrid HLA class I: 73.30 ± 3.36%) (Figures 2A,B). Moreover, similar results were obtained from DCs stimulated with each of the PLGA-chCPAp-MPLA, PLGA-chH1p-MPLA, and PLGA-chKMP-11p-MPLA nanoformulations (Figure S2 in Supplementary Material) or each of the p8-PLGA-chCPAp, p8-PLGA-chH1p, and p8-PLGA-chKMP-11p nanoformulations (Figure S3 in Supplementary Material), in comparison to DCs stimulated with each of PLGA-chCPAp, PLGA-chH1p, and PLGA-chKMP-11p nanoformulations, respectively. It is noteworthy that the significant (*p* < 0.001) increase observed in the number of DCs expressing the hybrid HLA-A2.1 molecule confirmed the successful design of the chimeric peptides to harbor T cell epitopes with high-binding affinity to HLA class I molecules.

**Figure 5:**
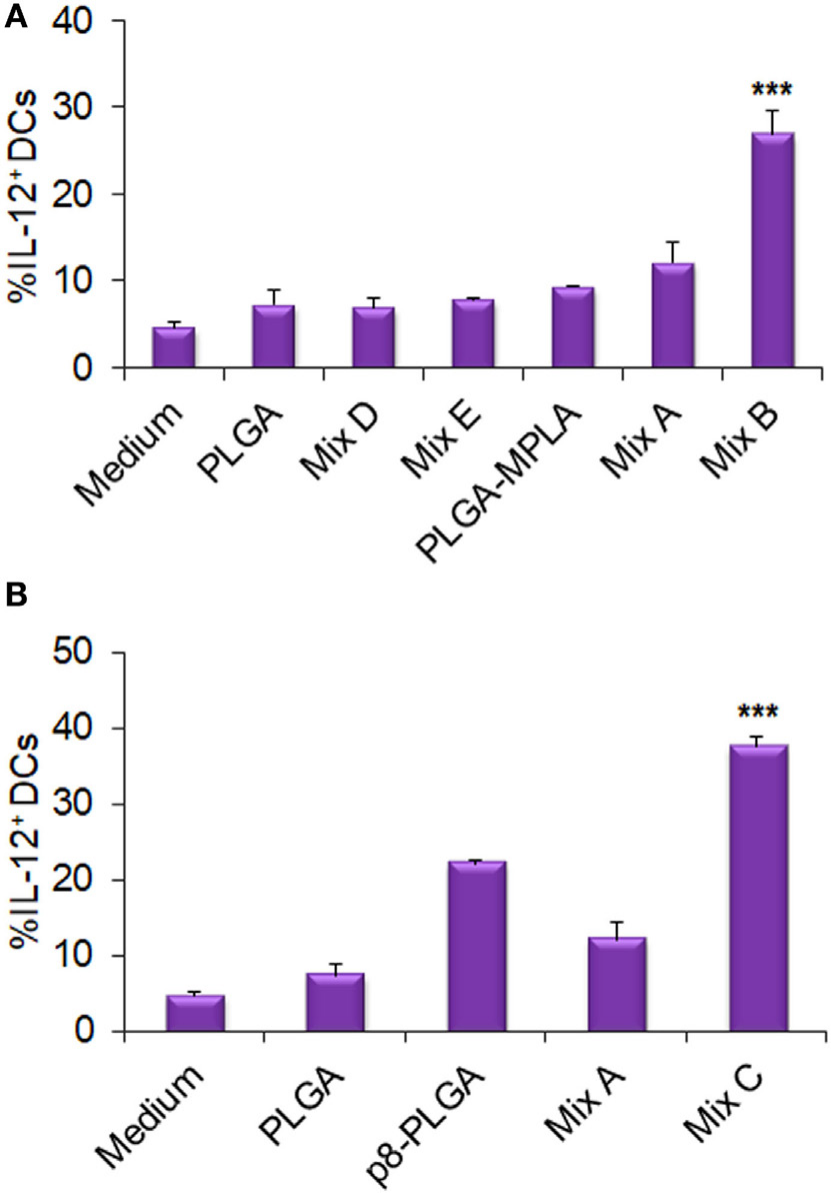
Functional differentiation of dendritic cells (DCs) stimulated with the differentially functionalized peptide-based poly(lactic-*co*-glycolic) acid (PLGA) nanoformulations in terms of IL-12 production. DCs were cultured for 24 h upon stimulation with the mix of PLGA-chCPAp, PLGA-chH1p, and PLGA-chKMP-11p nanoformulations (mix A), the mix of PLGA-chCPAp-MPLA, PLGA-chH1p-MPLA, and PLGA-chKMP-11p-MPLA nanoformulations (mix B), the mix of p8-PLGA-chCPAp, p8-PLGA-chH1p, and p8-PLGA-chKMP-11p nanoformulations (mix C), the mix of soluble chimeric peptides (mix D), and the mix of soluble chimeric peptides plus MPLA (mix E). DCs cultured in medium alone were used as negative control. DCs were also stimulated with empty PLGA nanoparticles (NPs), PLGA NPs with MPLA encapsulated, and PLGA NPs surface modified with p8 as control groups. The diagrams indicate the effect of MPLA incorporation **(A)** or the effect of surface modification with p8 **(B)** in the percentage of DCs producing IL-12. Results are expressed as the mean ± SD of three independent experiments. Significant differences between DCs stimulated with the mix B or the mix C and DCs stimulated with the mix A are indicated by ***p < 0.001.

Furthermore, as demonstrated in Figure 5, stimulation of DCs with the mix B (Figure 5A) or the mix C (Figure 5B) resulted in a significant increase of DCs that intracellularly produced IL-12 (26.94 ± 2.74 and 37.60 ± 1.43, respectively, vs. 12.16 ± 2.20%, *p* < 0.001), compared to DCs stimulated with the mix A. This observation supports the functional differentiation of DCs toward DC1 type. The high percentage of DCs that produced IL-12 (22.11 ± 0.53%) in response to p8-PLGA NPs indicated a role of the synthetic octapeptide in this biological process.

### DCs Stimulated with the Mix of PLGA Nanoformulations with MPLA Incorporation or Surface Modification Promoted Allogeneic T Cell Proliferation and IFNγ Production by CD4^+^ and CD8^+^ T Cells

**Figure 6:**
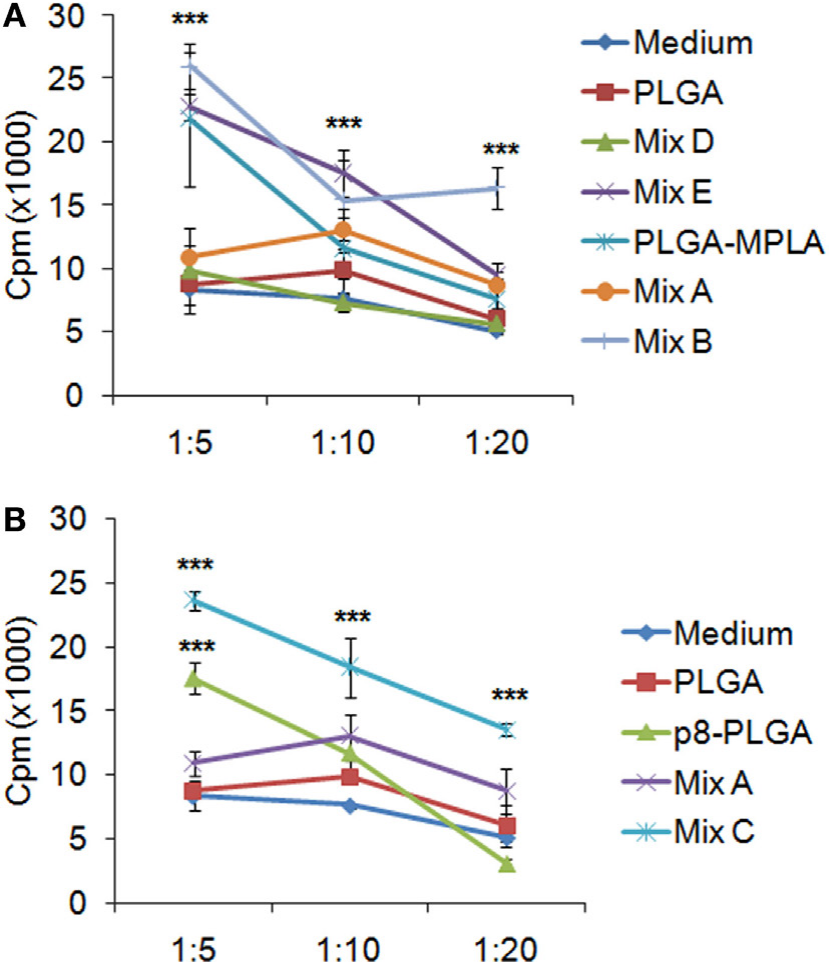
T cell priming induced by dendritic cells (DCs) stimulated with the differentially functionalized peptide-based poly(lactic-*co*-glycolic) acid (PLGA) nanoformulations. Spleen cells from naïve BALB/c mice were primed *in vitro* by DCs stimulated for 24 h with the mix of PLGA-chCPAp, PLGA-chH1p, and PLGA-KMP-11p nanoformulations (mix A), the mix of PLGA-chCPAp-MPLA, PLGA-chH1p-MPLA, and PLGA-KMP-11p-MPLA nanoformulations (mix B), the mix of p8-PLGA-chCPAp, p8-PLGA-chH1p, and p8-PLGA-KMP-11p nanoformulations (mix C), the mix of soluble chimeric peptides (mix D), and the mix of soluble chimeric peptides plus MPLA (mix E). Cultures were pulsed for the last 18 h with 1 μCi of ^3^[H]-TdR, and the proliferative potential was measured by ^3^[H]-TdR incorporation. The diagrams indicate the effect of MPLA incorporation **(A)** or the effect of surface modification with p8 **(B)** in the capacity of DCs stimulated with the mix B or the mix C to induce T cell priming. Samples were run in triplicates. Results are depicted as cpm ± SD from three independent experiments. Significant differences are indicated by ***p < 0.001.

Since the peptide-based PLGA nanoformulations and especially those with MPLA incorporation or surface modification proved capable to induce DCs maturation and IL-12 production, mixed leukocyte cultures were performed in order to investigate the capacity of these DCs to promote *in vitro* T cell proliferation. For this purpose, DCs stimulated with the peptide-based PLGA nanoformulations were co-cultured with allogeneic spleen cells at different stimulator/responder ratios and their proliferative potential was determined by ^3^[H]-TdR incorporation. The results obtained indicated that the optimum stimulator/responder ratio was that of 1:5. As shown in Figure 6, DCs stimulated with the mix of PLGA-chCPAp, PLGA-chH1p, and PLGA-chKMP-11p nanoformulations (mix A) proved able to promote T cell proliferation (cpm: 10,895 ± 953) compared to unstimulated DCs stimulated (cpm: 8,330 ± 1,167). However, the incorporation of the MPLA adjuvant, as well as the surface modification with p8, remarkably enhanced the competency of stimulated DCs to efficiently present the processed antigenic peptides to T cells. In more details, DCs stimulated with the mix of PLGA-chCPAp-MPLA, PLGA-chH1p-MPLA, and PLGA-chKMP-11p-MPLA nanoformulations (mix B) or the mix of p8-PLGA-chCPAp, p8-PLGA-chH1p, and p8-PLGA-chKMP-11p nanoformulations (mix C) triggered significantly increased lymphoproliferative responses in comparison to unstimulated DCs (cpm mix B: 26,014 ± 1,828 vs. 8,330 ± 1,167, *p* < 0.001; cpm mix C: 23,626 ± 776 vs. 8,330 ± 1,167, *p* < 0.001). It is noteworthy that DCs stimulated with PLGA-MPLA or p8-PLGA also induced T cell proliferation at significant levels compared to unstimulated DCs (*p* < 0.001) at the ratio 1:5. This difference was not observed at ratio 1:20 in contrast to DCs stimulated with mix B or mix C. Spleen cells cultured in medium alone and *in vitro* stimulated with the mitogen ConA were used as positive control of proliferation (cpm: 40,118 ± 1,936, data not shown).

**Figure 7:**
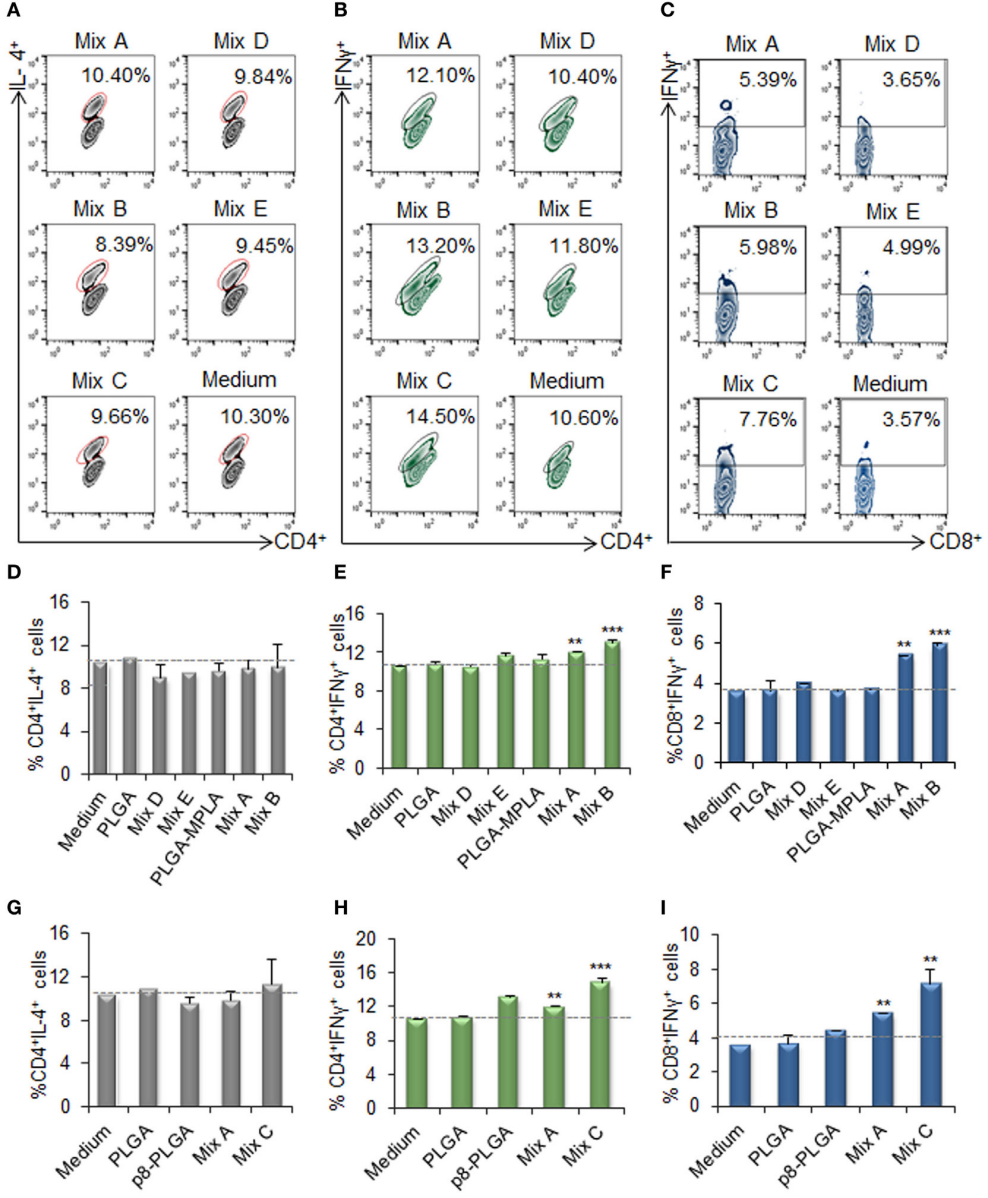
IFNγ-producing CD4^+^ and CD8^+^ T cells among the spleen cell populations primed by dendritic cells (DCs) stimulated with the mix B or C. Spleen cells from naïve BALB/c mice were primed *in vitro* by DCs stimulated for 24 h with the different mixes of differentially functionalized nanoformulations (mix A, mix B, and mix C), the mix of soluble chimeric peptides (mix D), and the mix of soluble chimeric peptides plus monophosphoryl lipid A (MPLA) (mix E). Then, spleen cells were stained and analyzed by flow cytometry. The representative contour plots depict IL-4-producing CD4^+^ T cells **(A)**, and IFNγ-producing CD4^+^ **(B)**, and CD8^+^ **(C)** T cells. Diagram showing the percentage of **(D,G)** IL-4-producing CD4^+^ T cells, **(E,H)** IFNγ-producing CD4^+^, and **(F,I)** IFNγ-producing CD8^+^ T cells. Results are expressed as the mean ± SD and significant differences between T cells activated by DCs stimulated with mix A, mix B or mix C and T cells activated by unstimulated DCs are indicated by **p < 0.01, or ***p < 0.001.

Flow cytometry analysis was conducted to unveil the presence of CD4^+^ T cells producing IL-4 (indicative of T_H2_ polarization) or IFNγ (indicative of T_H1_ polarization), as well as IFNγ-producing CD8^+^ T cells. Flow cytometry results revealed no increase in the population of IL-4-producing CD4^+^ T cells in comparison to T cells co-cultured with unstimulated DCs (Figures 7A,D,G). On the contrary, a significant increase was observed in the populations of IFNγ-producing CD4^+^ T cells (Figures 7B,E,H) and CD8^+^ T cells (Figures 7C,F,I) upon activation by DCs stimulated with the mix of PLGA-chCPAp, PLGA-chH1p, and PLGA-chKMP-11p (Mix A; CD4^+^ T cells: 12.0 ± 0.14%, *p* < 0.01 and CD8^+^ T cells: 5.4 ± 0.05%, *p* < 0.01) compared to unstimulated DCs. A further increase in IFNγ-producing CD4^+^ and CD8^+^ T cells was observed upon activation with DCs stimulated with PLGA-chCPAp-MPLA, PLGA-chH1p-MPLA, and PLGA-chKMP-11p-MPLA nanoformulations (mix B; CD4^+^ T cells: 13.0 ± 0.28%, *p* < 0.001 and CD8^+^ T cells: 5.88 ± 0.14%, *p* < 0.001) or the mix of p8-PLGA-chCPAp, p8-PLGA-chH1p, and p8-PLGA-chKMP-11p nanoformulations (mix C; CD4^+^ T cells: 14.85 ± 0.49%, *p* < 0.001 and CD8^+^ T cells: 7.16 ± 0.86%, *p* < 0.01).

### Transcriptome Analysis of DCs Stimulated with the Differentially Functionalized PLGA Nanoformulations Revealed that MPLA Incorporation Induced the Most Robust Transcriptional Activation

**Figure 8:**
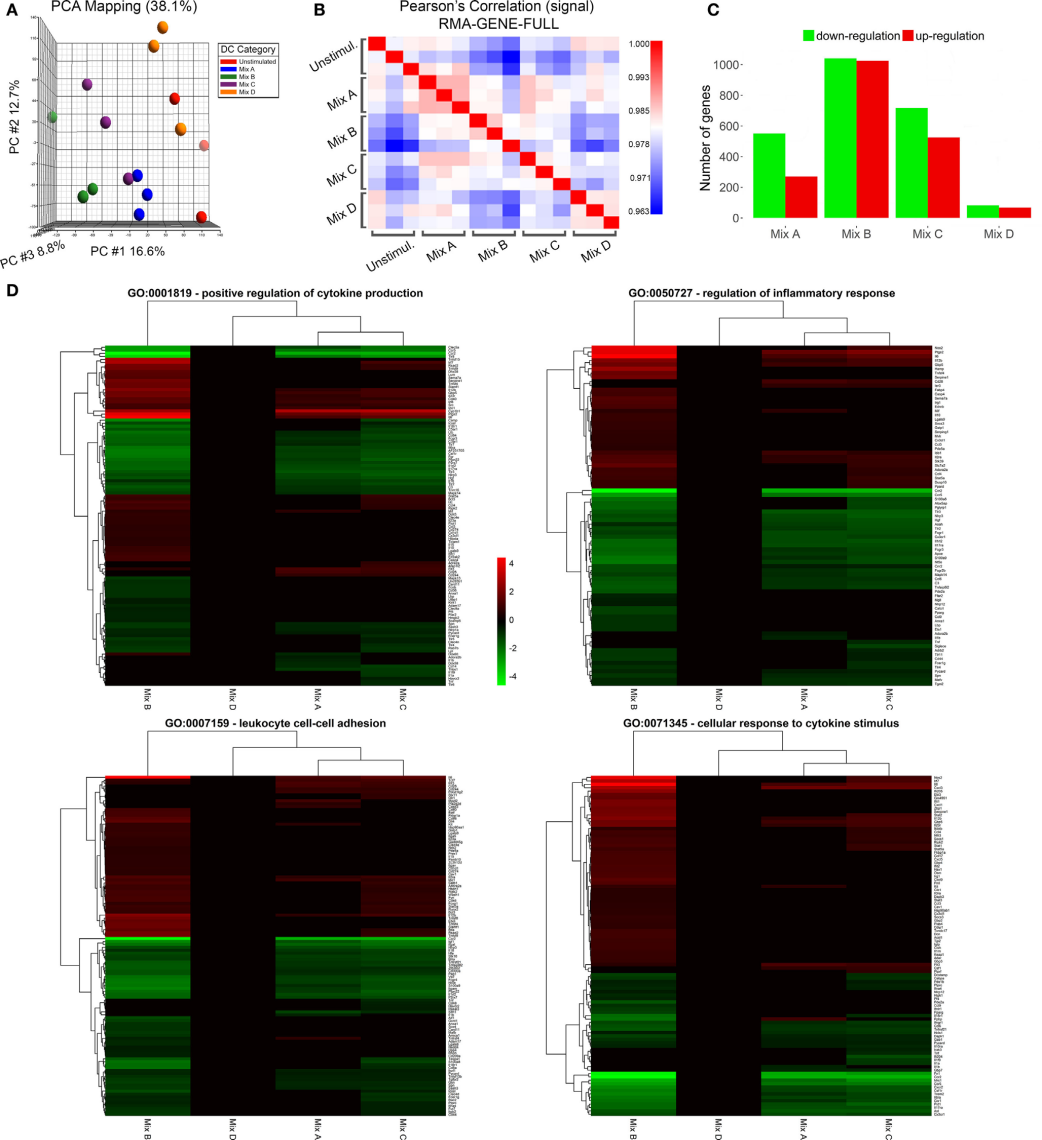
Microarray sample quality control and differential expression analysis. **(A)** Principal component analysis clustering of microarray samples and **(B)** sample-sample correlation heat map depicting relationship between replicates and/or samples. **(C)** Barplot of differentially up- and downregulated genes of dendritic cells (DCs) stimulated with the mix of poly(lactic-*co*-glycolic) acid (PLGA)-chCPAp, PLGA-chH1p, and PLGA-KMP-11p nanoformulations (mix A), the mix of PLGA-chCPAp-monophosphoryl lipid A (MPLA), PLGA-chH1p-MPLA, and PLGA-KMP-11p-MPLA nanoformulations (mix B), the mix of p8-PLGA-chCPAp, p8-PLGA-chH1p, and p8-PLGA-KMP-11p nanoformulations (mix C), or the mix of soluble chimeric peptides (mix D) vs. unstimulated DCs. **(D)** Heatmap representations of differentially expressed genes included in GO terms GO:0001819—positive regulation of cytokine production, GO:0050727—regulation of inflammatory response, GO:0007159—leukocyte cell–cell adhesion and GO:0071345—cellular response to cytokine stimulus. Instances with −0.585 < log2(fold change) < 0.585 and/or *p*-value >0.05 significance threshold are not colored. A *p*-value threshold of 0.05 and a log2 (fold change) threshold of ±0.585 (at least1.5-fold change) was used to determine differentially expressed genes of samples Mix A–D vs. unstimulated DCs.

In an attempt to unveil changes in the gene-expression profile of DCs stimulated with the differentially functionalized PLGA nanoformulations for 18 h, transcriptome analysis was performed using microarrays. PCA and Pearson correlation analysis of samples displayed satisfactory replicate resemblance (Figures 8A,B). Microarray data analysis revealed considerable differences in the gene-expression profiles of DCs stimulated with the different mixes of peptide-based PLGA nanoformulations or the mix of soluble chimeric peptides (Figures 8C,D). The highest number of differentially expressed genes was observed in DCs stimulated with the PLGA-chCPAp-MPLA, PLGA-chH1p-MPLA, and PLGA-chKMP-11p-MPLA nanoformulations (mix B, 2,104 differentially expressed genes; 1,027 up- and 1,077 downregulated), followed by DCs stimulated with the p8-PLGA-chCPAp, p8-PLGA-chH1p, and p8-PLGA-chKMP-11p nanoformulations (mix C, 1,273 differentially expressed genes; 527 up- and 746 downregulated), and then DCs stimulated with the PLGA-chCPAp, PLGA-chH1p, and PLGA-chKMP-11p nanoformulations (mix A, 847 differentially expressed genes; 278 up- and 569 downregulated). Importantly, much milder differences were observed for DCs stimulated with the mix of soluble chimeric peptides (mix D, 191 differentially expressed genes; 69 up- and 122 downregulated) compared to unstimulated DCs. Therefore, the hierarchy of potency in inducing gene-expression changes was mix B > mix C > mix A > mix D, stressing that the overall magnitude of activation was much higher in DCs stimulated with the mix B.

**Figure 9:**
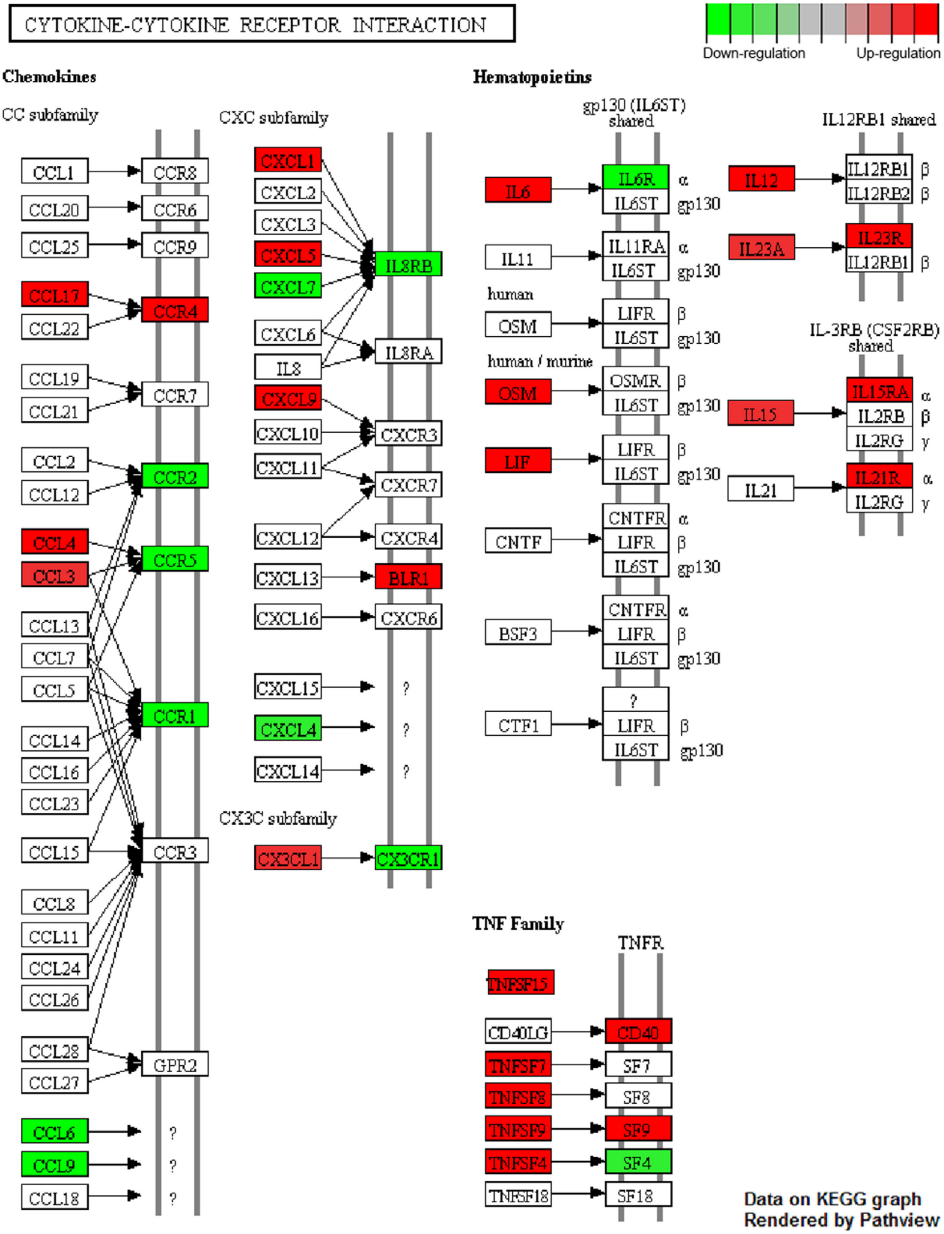
Cytokine–cytokine receptor interaction pathway. Genes found to be differentially up- (red) and downregulated (green) in dendritic cells (DCs) stimulated with the mix of PLGA-chCPAp-MPLA, PLGA-chH1p-MPLA, and PLGA-KMP-11p-MPLA nanoformulations (mix B) compared to unstimulated DCs. “Cytokine-cytokine receptor interaction” (mmu04060) KEGG pathway was adapted in order to place emphasis specifically on the deregulated genes (*p* < 0.05, 1.5-fold upregulation or downregulation), as dictated by the microarray experiments. Pathview package was utilized to color nodes of differentially expressed genes.

Enrichment analysis of differentially expressed genes for GO terms (Biological Processes) revealed that DCs stimulated with the mix B or the mix C exhibited a higher number of significantly enriched terms compared to DCs stimulated with the mix A, while no significant enrichment was observed in the case of DCs stimulated with the mix D. More specifically, DCs stimulated with the mix B or the mix C shared common genes involved in cytokine production (GO:0001819), inflammatory response (GO:0050727), leukocyte cell–cell adhesion (GO:0007159), and cellular response to cytokine stimulus (GO:0071345), as well as in other biological processes related to antigen processing and presentation or adaptive immunity (Data Sheet S1 in Supplementary Material). Focusing on a number of the most significantly enriched GO terms (Figure 8D; Table 4), genes encoding pro-inflammatory cytokines, such as *Il12b, Il6*, type-I, and II IFN-response elements (*Irf7, Stat5a, Irf8, Gbp5, Ido1, Stat1, Ifi205*, etc.) and DCs maturation markers (*Cd40*) were upregulated in both groups. However, a much higher number of upregulated genes involved in the above biological processes were found to be specifically expressed in DCs stimulated with the mix B. Between these specifically upregulated genes, they were identified IFN-response genes (*Ifih1, Ifit1, Ifit2, Iigp1, Irg1, Gbp2*, and *Gbp4*), inflammation-related genes (*Il10, Il23a, Il15*, and *Saa3*), and genes encoding chemokines that act to recruit leukocytes (*Ccl3, Cx3cl1, Mif*, and *Cxcl1*). Moreover, a remarkable number of genes was identified characteristic for the type 1 DCs phenotype that could induce a subsequent CD8^+^ T cell activation (*Psmb10, Hsp90aa1, Hsp90ab1, Igtp, Ccl3, Dhx58, Tnfsf8, Il15, Olr*, etc.) and CD4^+^ T_H1_ polarization (*Cd86, Cish, Dll4, Cxcl9, Cx3cl1, Osm, Il1f6, Tnfsf15, Kit, Clec4e*, etc.) (Table 4; Figure 9). Overall, the above findings suggested that DCs exposed to mix B could probably be in a more advanced state of maturity and functional differentiation and might be able to induce specific T cell responses.

**Table 4:** Unique or common upregulated genes in dendritic cells stimulated with mix B or mix C that enriched GO terms related to immune response.

	Mix B		Mix C		Genes in common
	FDR	Unique genes	FDR	Unique genes	
GO:0001819	5.28E−27	*Ddx60, Lgals9, Dhx58, Il1f6, Clec4e, Tnfsf15, Il10, Eif2ak2, Ifih1, Il23a, Serpine1, Tnfsf4, Ticam1, Ddit3, Flot1, Lum, Sema7a, Tlr1, Cd1d1, Il15, Casp4, Cd274, Hilpda, Ripk2, Slamf1, Ccl3, Cx3cl1, Mif*	8.77E−22	*Adra2a, Afap1l2, F2rl1, Cd244, Cd28*	*Irf7, Rsad2, Hc, Bcl3, Stat5a, Ccl4, Src, Tnfsf9, Il12b, Il23r, Cd40, Irf8, Ido1, Gbp5, Il6, Flt3, Ptgs2, Cyp1b1*
Positive regulation of cytokine production					

GO:0050727	2.29E−20	*Lgals9, Il1r1, Serping1, Fabp4, Il10, Ednrb, Serpine1, Tnfsf4, Gstp1, Hamp, Sema7a, Socs3, Casp4, Irg1, Ccl3, Cx3cl1, Mvk, Pde5a, Tnfaip6, Mif*	4.55E−16	*Ier3, Cd28*	*Slc7a2, Nos2, Stat5a, Adora2a, Ccl4, Ppard, Il12b, Il2ra, Stk39, Dusp10, Ido1, Gbp5, Il6, Ptgs2*
Regulation of inflammatory response					

GO:0007159	7.91E−18	*Lgals9, Batf, Cav1, Itgav, Clec4e, Cd80, Itga5, Gadd45g, Tnfsf8, Prex1, Il23a, Tnfsf4, Dll4, Gstp1, Ebi3, Cd1d1, Il15, Fkbp1a, Cd274, Psmb10, Ripk2, Slamf1, Nck2, Cd86, Pde5a, Hsp90aa1, Zc3h12d, Kit*	1.19E−15	*Olr1, Stx11, F2rl1, Cd244, Pdcd1lg2, Cd28*	*Rsad2, Hsph1, Cdk6, Btla, Foxp1, Satb1, Fyn, Bcl3, Stat5a, Adora2a, Runx2, Wash1, Tnfsf9, Il12b, Il2ra, Ido1, Tcf7, Il6, Flt3*
Leukocyte cell–cell adhesion					

GO:0071345	3.25E−17	*Gm4951, Ifit1, Ifit2, Saa3, Igtp, Zbp1, Cav1, Il1r1, Pml, Il1f6, Iigp1, Dcn, Cxcl9, Adar, Osm, Acsl1, Keap1, Serpine1, Pyhin1, Hsp90ab1, Il1rn, Gbp4, Il3ra, Ebi3, Dapk3, Cxcl5, Socs3, Fkbp1a, Gbp3, Hax1, Pias4, Tjp2, Cish, Cib1, Irg1, Ripk2, Cxcl1, Stat3, Cdip1, Ccl3, Cx3cl1, Gbp2, Ccl17, Gbp2b, Kit*	1.91E−09	*Ptprf, F2rl1, Csf1*	*Irf7, Ifi205, Nos2, Nfil3, Stat2, Ikbkb, Socs1, Stat1, Stat5a, Ccl4, Il12b, Txndc17, Il23r, Gbp5, Il6, Flt3, Cxcl3*
Cellular response to cytokine stimulus					

### Immunization of HLA A2.1 Transgenic Mice with the Mix of Peptide-Based PLGA Nanoformulations with MPLA Incorporation Promoted Peptide-Specific IFNγ-Producing CD8^+^ T Cell Populations

**Figure 10:**
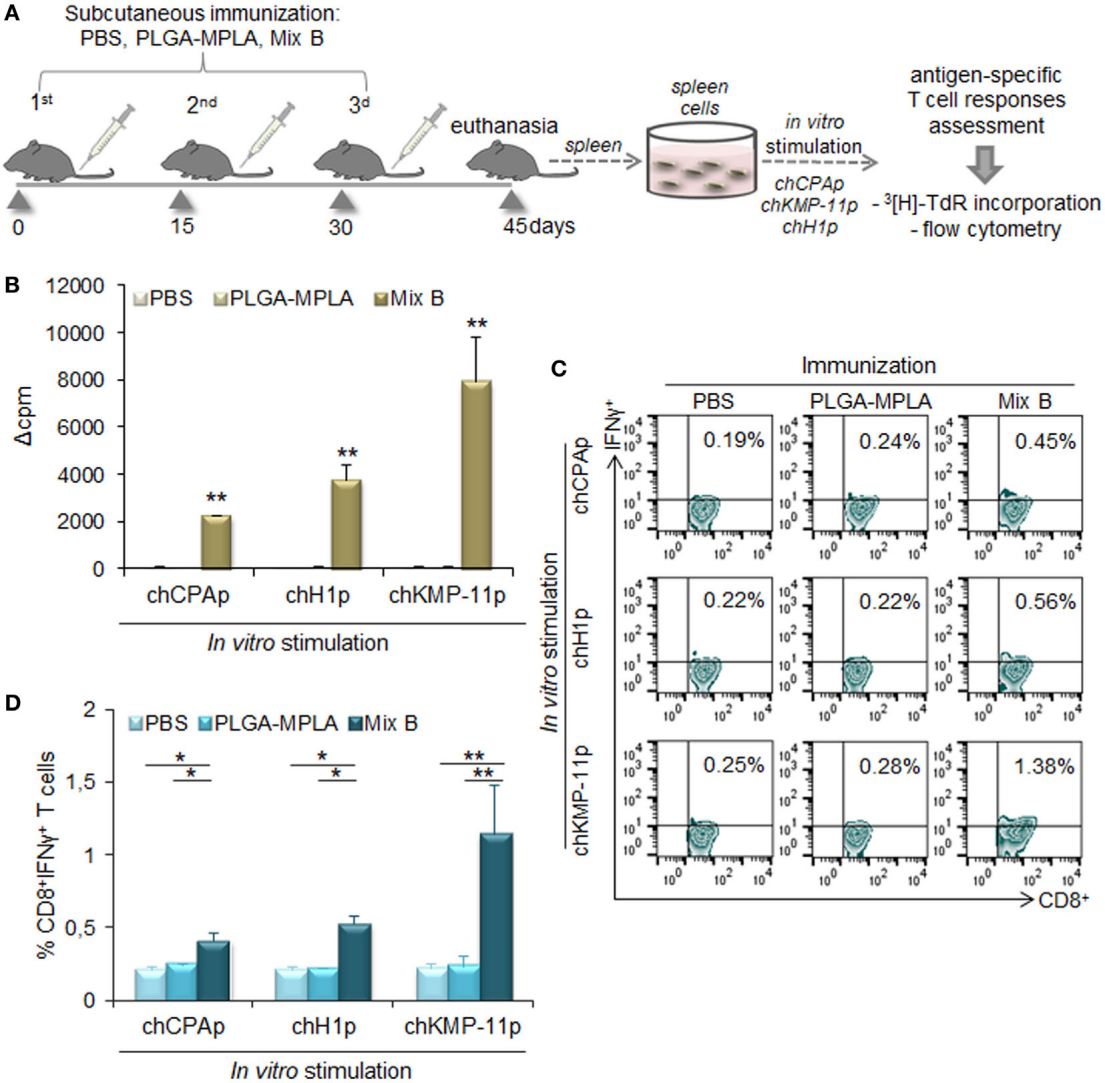
Immunization with the mix of peptide-based poly(lactic-*co*-glycolic) acid (PLGA) nanoformulations with monophosphoryl lipid A (MPLA) incorporation induced peptide-specific T cell responses in HLA A2.1 transgenic mice. **(A)** Immunization scheme. Mice were subcutaneously immunized with the mix of PLGA-chCPAp-MPLA, PLGA-chH1p-MPLA, and PLGA-KMP-11p-MPLA nanoformulations (mix B), PLGA-MPLA nanoformulations or only PBS. Two weeks after the last boosting dose, the spleen of each mouse was aseptically removed and spleen cells were stimulated *in vitro* with each of the chimeric peptides (chCPAp, chH1p, and chKMP-11p) in order to assess induced peptide-specific T cell responses. **(B)** Peptide-specific T cell expansion was measured by ^3^[H]-TdR incorporation. Samples were run in triplicates. Results are represented as Δcpm ± SD (cpm of stimulated spleen cells—cpm of unstimulated spleen cells), and significant differences are indicated by **p < 0.01. **(C,D)** *In vitro*-stimulated spleen cells were stained with fluorochrome-labeled anti-CD3, anti-CD8, and anti-IFNγ monoclonal antibodies and analyzed with flow cytometry. **(C)** The representative contour plots depict the peptide-specific IFNγ-producing CD8^+^ T cells. **(D)** The diagrams show the percentage of peptide-specific IFNγ-producing CD8^+^ T cells. Results are expressed as the mean ± SD and significant differences between spleen cells from mice immunized with mix B and spleen cells from mice immunized with PLGA-MPLA or PBS are indicated by *p < 0.05 or **p < 0.01.

The *in vitro* screening of the differentially functionalized PLGA nanoformulations in DCs gave precedence to the mix of PLGA-chCPAp-MPLA, PLGA-chH1p-MPLA, and PLGA-chKMP-11p-MPLA nanoformulations (mix B) to be evaluated *in vivo* in terms of immunogenicity, as a promising peptide-based nanovaccine. For this purpose, HLA A2.1 transgenic mice were subcutaneously injected with the mix B and boosted twice in a 2-week interval (Figure 10A). The specific T cell expansion induced by each of the chimeric peptides was initially assessed by ^3^[H]-TdR incorporation. As shown in Figure 10B, chCPAp, chH1p, and chKMP-11p induced proliferation of spleen cells from mice immunized with mix B compared to spleen cells from non-immunized mice (received only PBS), upon *in vitro* stimulation. More specifically, chKMP-11p induced the strongest proliferation (Δcpm: 7,908 ± 1,886 vs. 65 ± 20, *p* < 0.01), followed by chH1p (Δcpm: 3,764 ± 643 vs. 42 ± 6, *p* < 0.01) and chCPAp (Δcpm: 2,232 ± 112 vs. 66 ± 5, *p* < 0.05). Mice immunized with PLGA-MPLA nanoformulations did not show specific T cell expansion. Spleen cells cultured in medium alone and *in vitro* stimulated with the mitogen ConA were used as positive control of proliferation (Δcpm: 39,726 ± 3,061, data not shown).

Flow cytometry analysis was then performed to detect peptide-specific IFNγ-producing CD8^+^ T cell populations (Figures 10C,D). The highest number of peptide-specific IFNγ-producing CD8^+^ T cells was observed upon *in vitro* stimulation with chKMP-11p of spleen cells from mice immunized with mix B compared to spleen cells from non-immunized mice (1.34 ± 0.35 vs. 0.22 ± 0.03%, *p* < 0.05). Chimeric peptides chH1p and chCPAp were also able to stimulate specific IFNγ-producing CD8^+^ T cells to a lower level (0.53 ± 0.05 vs. 0.21 ± 0.02%, *p* < 0.01 and 0.41 ± 0.06 vs. 0.20 ± 0.02%, *p* < 0.05, respectively). It must be also noted that spleen cells from mice immunized with PLGA-MPLA nanoformulations did not exhibit IFNγ-producing CD8^+^ T cell populations, similar to spleen cells from non-immunized mice.

### Immunization of HLA A2.1 Transgenic Mice with the Mix of Peptide-Based PLGA Nanoformulations with MPLA Incorporation Conferred Protection against *L. infantum* Infection

**Figure 11:**
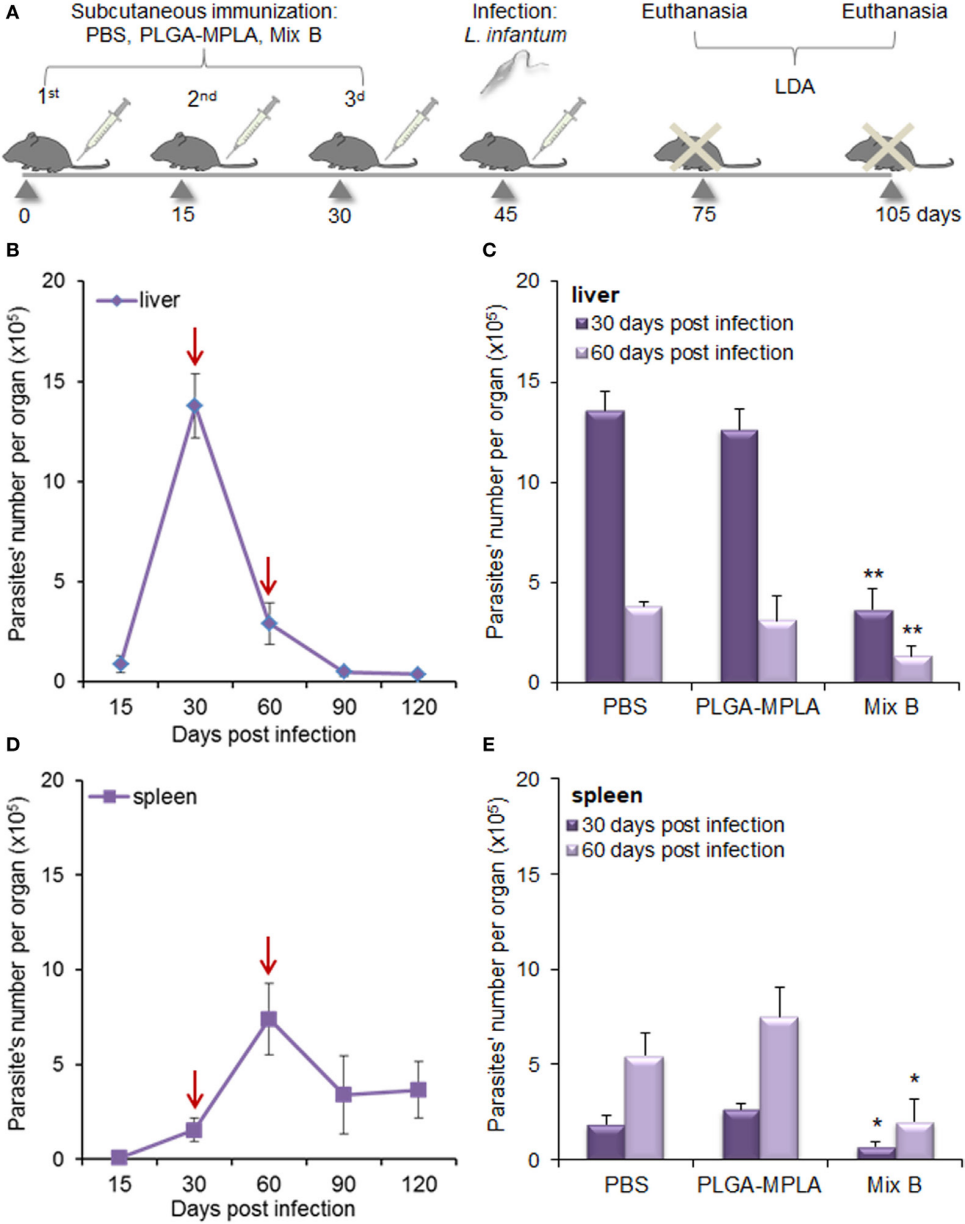
Immunization with the mix of peptide-based poly(lactic-*co*-glycolic) acid (PLGA) nanoformulations with monophosphoryl lipid A (MPLA) incorporation conferred significant protection against *Leishmania infantum* infection in HLA A2.1 transgenic mice. **(A)** Immunization and infection scheme. Mice were subcutaneously immunized with the mix of PLGA-chCPAp-MPLA, PLGA-chH1p-MPLA, and PLGA-KMP-11p-MPLA nanoformulations (mix B), PLGA-MPLA nanoformulations or only PBS. Two weeks after the last boosting dose, immunized and non-immunized mice were infected intravenously with 2 × 10^7^ *L. infantum* promastigotes and parasite burden was evaluated 1 and 2 months post-infection with a limiting dilution assay. **(B,D)** Time course of infection in liver **(B)** and spleen **(D)** of HLA A2.1 transgenic mice infected with *L. infantum* promastigotes. Red arrows indicate the time points selected for the evaluation of immunization’s protective effect. **(C,E)** Parasite burden in the liver **(C)** and the spleen **(E)** of immunized and non-immunized mice 1 and 2 months post-infection with *L. infantum*. Results are presented as the mean ± SD of five individual mice per group and significant differences between the parasite burden of immunized with mix B mice and the parasite burden of non-immunized mice are indicated by *p < 0.05 or **p < 0.01.

In order to investigate whether the peptide-specific T cell responses observed in HLA A2.1 transgenic mice immunized with the mix of PLGA-chCPAp-MPLA, PLGA-chH1p-MPLA, and PLGA-chKMP-11p-MPLA nanoformulations (mix B) could confer protection against *L. infantum* infection, immunized and non-immunized mice were infected intravenously with *L. infantum* promastigotes and the parasite burden was assessed in liver and spleen by a limiting dilution assay 1 and 2 months post-infection (Figure 11A). According to the infection kinetics in HLA A2.1 transgenic mice, the parasite burden reached a peak at 1 month post-infection in the liver (Figure 11B) and at 2 months post-infection in the spleen (Figure 11D), followed by a decrease in both organs. The results obtained indicated that immunization with the mix B reduced significantly hepatic and splenic parasite burden 1 month post-infection by 72.81% (*p* < 0.01, Figure 11C) and 61.98% (*p* < 0.05, Figure 11E), respectively, compared to the non-immunized control group. Assessment of the parasite burden 2 months post-infection revealed a maintenance of the protective effect with 64.4% reduction of parasite burden in the liver (*p* < 0.01, Figure 11C) and 73.64% reduction of parasite burden in the spleen (*p* < 0.05, Figure 11E). It also must be noted that immunization with PLGA-MPLA nanoformulations did not confer protection against *L. infantum* infection, indicating that the protective effect observed in liver and spleen of immunized with the mix B HLA A2.1 transgenic mice was peptide specific.

## Discussion

Immunoinformatics analyses based on algorithms that predict with high accuracy immunodominant epitopes on protein antigens could greatly enhance “polytope vaccine” design and development against infectious diseases, such as VL, since the most efficient immune response to pathogens is derived from different T cells that respond to an ensemble of pathogen-derived specific epitopes (47). Different research groups have focused on MHC class I- and/or MHC class II-restricted epitope prediction from *Leishmania* spp. vaccine candidates such as A2, GP63, KMP-11, CPs, LmsTI-1, TSA, LeIF, and LPG-3 with the view of a peptide-based vaccine generation against CL or VL (36, 48–51). Following this promising approach, in a previous study, we performed an *in silico* analysis of the *L. infantum* proteins CPA, histone H1, and KMP-11 in order to identify T cell epitopes presented on mouse and human MHC class I and II molecules and design multi-epitope peptides that were validated in terms of immunogenicity in BALB/c mice (29). Among these multi-epitope peptides, CPA_p2, CPA_p3, H1_p1, and H1_p3 proved capable to induce a T cell response characterized by CD8^+^ and CD4^+^ T cell priming with IFNγ production in immunized mice, whereas KMP-11_p1 abrogated the secretion of IL-10.

The *in silico* analysis of the three *L. infantum* proteins also revealed a remarkable number of binding epitopes to HLA A*0201 allele. Among HLA A2 allelic variants, HLA A*0201 is the most prevalent and common in all ethnic groups, hence its peptide-binding motif is commonly used for epitope prediction of proteins from viruses such as hepatitis B and C viruses, human immunodeficiency virus, Epstein–Barr virus, human papillomavirus (43), as well as *Leishmania* parasites (51, 52). All the multi-epitope peptides contained more than one HLA A*0201-restricted epitopes and were used for the design of longer chimeric peptides, with the use of appropriate linkers. Such an approach can help to overcome the fact that peptides shorter than 30 amino acids long may bind directly to MHC class I or II molecules of non-professional antigen-presenting cells, thereby potentially stimulating tolerance or anergy (53, 54).

Linkers have a pivotal role in functional and structural features of a peptide-based vaccine, since tandem fusion of peptides may result in generation of a new “protein” with novel characteristics and potent loss of the predicted MHC class I- or II-restricted epitopes. Linkers starting with alanine are more frequently used when targeting CD8^+^ T cell responses, as the chance of proteasomal cleavage increases once the first amino acid next to the C-terminal peptide is alanine (55). Thus, the multi-epitope peptides of CPA (CPA_p2 and CPA_p3) and histone H1 (H1_p1 and H1_p3) were linked together by AAY, a short amino acid motif that is documented to support epitope generation and has been used in several studies on epitope vaccine design against cancer or infectious diseases (56–59). The two alanine residues flanking the C-terminal support C-terminal cleavage of the epitope without negatively influencing the N-terminal cleavage of the adjacent epitope (60), and further the generation of the C-terminal provides a suitable site for binding to TAP transporter or other chaperons (61).

KMP-11 derived multi-epitope peptide (KMP-11_p1) was used in conjunction with the pan DR epitope (PADRE), a universal synthetic 13 aa peptide with high-binding affinity to 15 of the 16 most common HLA-DR molecules that is specifically engineered to be immunogenic in humans (62). CD4^+^ T cells are well documented to play a central role in priming and maintenance of CD8^+^ T cell effector functions, and thus PADRE could be considered a crucial component of prophylactic or immunotherapeutic vaccines against tumors and intracellular pathogens. Indeed, PADRE has been shown to augment the potency of vaccines designed to stimulate a cellular immune response (62). Multi-epitope peptides targeting CD8^+^ T cell responses in conjunction with PADRE and a signaling peptide proved capable to increase memory CD8^+^ T cells producing IFNγ and elicit protective immunity in transgenic mice challenged with *Toxoplasma gondii* (63). In another study, the use of PADRE in combination with a synthetic multi-epitope peptide derived from the tumor-associated antigen Her2/neu improved vaccine potency in terms of IFNγ producing CD4^+^ and CD8^+^ T cells expansion in mice (64). PADRE and KMP-11_p1 were fused together *via* the linker HEYGAEALERAG that provides the specific cleavage target for both proteasomal and lysosomal degradation system enhancing epitope presentation (57). This 12 aa peptide consists of five appropriate cleavage sites Y3-G4, A5-E6, A7-L8, L8-E9, and R10-A11 specified for eukaryotic proteasome complexes in which A5-E6 is superior cleavage site (65) and has been used in a number of studies on multi-epitope peptide-based vaccine strategy combating cancer and intracellular infectious agents (57, 66, 67).

Antigen presentation is a crucial step in the initiation of an effective immune response and DCs own the unique ability to efficiently present processed antigenic peptides to T cells in the context of MHC class I or II molecules, playing a pivotal role in the orchestration of the adaptive immune response. Peptides are weak immune stimulators, and proper particulate delivery systems and/or adjuvants are needed to enhance their immunogenicity providing efficacious targeting of DCs. Thus, each chimeric peptide was synthesized and encapsulated in PLGA NPs (~300 nm) alone or in combination with MPLA adjuvant, or in PLGA NPs surface modified with the octapeptide p8 aiming their effective delivery to DCs. PLGA NPs are well suited for vaccine delivery due to the numerous advantages they offer including safety and endocytosis by DCs. A previous study has demonstrated that PLGA NPs 300 nm in average size exhibited low toxicity and were efficiently internalized by antigen-presenting cells—macrophages, B cells, and DCs—*in vitro* and *in vivo* (68). In the same study, PLGA NPs surface modified with p8 were proved to be internalized more efficiently by DCs *in vitro*.

This is the first report, to the best of our knowledge, describing the design and construction of an experimental nanovaccine against *L. infantum* infection based on a mix of PLGA NPs loaded with different synthetic long peptides from CPA, histone H1, and KMP-11 proteins, alone or in combination with the adjuvant MPLA, or a mix of PLGA NPs surface modified with the octapeptide p8 and loaded with the different synthetic long peptides. Since each chimeric peptide contained more than one HLA A2*0201-restricted epitopes, the effect of these peptide-based PLGA nanoformulations was examined in bone marrow-derived DCs isolated from transgenic mice expressing an interspecies hybrid MHC class I gene with the alpha-1 and alpha-2 domains of the human HLA-A2.1 gene, with the view of selecting the most promising nanovaccine candidate for *in vivo* evaluation. This transgenic strain enables the modeling of human T cell immune responses to HLA-A2.1 presented antigens and may be precious tool in hand to study the immunogenicity of *in silico* selected peptides for vaccination purposes. Many vaccine trials primarily reported as protective in wild-type animals exhibit a moderate efficacy in humans, partially due to the fact that human and animal MHC molecules may have different influence on the outcome of an immune response (69). Humanized transgenic mice expressing human HLA molecules have shown promising results despite subtle differences in the antigen-processing machinery including proteasome cleavage and TAP molecules affinity for peptides (70), since the immunological hierarchy is approximately the same in both models and about 80% of peptides immunogenic in one are also immunogenic in the other (71).

Apart from enhancing antigen uptake, the main rationale behind DCs targeting remains the efficient antigen presentation to CD4^+^ and CD8^+^ T cells that requires beforehand the development of a strong maturation profile. In this study, the encapsulation of chimeric peptides in PLGA NPs resulted in a significant increase in the number of DCs expressing the co-stimulatory molecules CD40, CD80, CD86, as well as the murine MHC class I and II molecules and the hybrid HLA-A2.1 molecule compared to DCs stimulated with the mix of soluble chimeric peptides. Previous studies have demonstrated that PLGA NPs loaded with soluble antigen or peptides strengthened DCs maturation in comparison to antigens in a soluble form or empty PLGA NPs (68, 72). Microarray data analysis strongly supported these findings, since DCs stimulated with the mix of soluble chimeric peptides exhibited a gene-expression profile similar to that of unstimulated DCs without enriched GO terms relevant to immune response. Both flow cytometry and microarray results demonstrated that MPLA incorporation in the peptide-based PLGA nanoformulations, as well as surface modification with p8 further enhanced DCs maturation. Particulate delivery of TLR ligands, such as MPLA adjuvant, offers several advantages over their administration in a soluble form. Delivery of TLR ligands in PLGA NPs would permit the use of very small doses and limit the non-specific immune activation and/or toxicity that may result upon systemic administration, as well as it may facilitate a sustained TLR signaling in DCs (73). A list of earlier studies have shown superior DCs and/or T cells activation when antigens were co-delivered in PLGA NPs with MPLA adjuvant (32, 68, 74–76). On the other hand, the attachment of targeting moieties on PLGA NPs surface cannot only facilitate the uptake by DCs, but can also enhance DCs maturation and ultimately lead to improving the effectiveness of vaccine formulation (73).

It is noteworthy that the significant increase in the number of DCs expressing the hybrid HLA-A2.1 confirms the achievement of antigen cross-presentation and the successful design of chimeric peptides to harbor epitopes that target mainly CD8^+^ T cell responses (Figures 2–4). PLGA NPs can escape from endosomes and extrude though endosomal membrane into the cytoplasm, where encapsulated antigens can be released, processed by the proteasome, and cross-presented by MHC class I molecules (73). In complement with the above findings, T cell proliferation assay and flow cytometry results demonstrated that both DCs stimulated with the mix of PLGA-chCPAp-MPLA, PLGA-chH1p-MPLA, and PLGA-chKMP-11p-MPLA nanoformulations (mix B) and DCs stimulated with the mix of p8-PLGA-chCPAp, p8-PLGA-chH1p, and p8-PLGA-chKMP-11p nanoformulations (mix C) were proved capable to activate CD8^+^ T cell populations with IFNγ production (Figure 7). Microarray data analysis provided robust evidence for the accuracy of the results obtained from flow cytometry, since it revealed upregulated genes (*Tap1, Tap2, Psme2, Tapbp*, etc.) related to antigen processing and presentation in the context of MHC class I molecules in both DCs stimulated with mix B or mix C (Data Sheet S1 in Supplementary Material). However, a number of genes exclusively upregulated in DCs stimulated with mix B indicated a greater potency of these peptide-based PLGA nanoformulations to promote CD8^+^ T cell responses (Data Sheet S1 in Supplementary Material; Table 4). Among them, *Psmb10* encodes the proteasome subunit beta type-10, a protein with major role in the immune system as part of an immunoproteasome formed by replacing constitutive beta subunits with inducible beta subunits that possess specific cleavage properties aiding in the release of peptides directed to MHC class I antigen presentation (77). *Hsp90aa1* and *Hsp90ab1* encode the two forms—inducible and constitutive, respectively—of the cytosolic heat shock protein 90 alpha, an endogenous chaperon that associates with the N-terminally extended peptides after proteasomal degradation (78) and is essential for cross-presentation of both soluble and cell-associated antigens by DCs (79). Interferon gamma induced protein 3 (IRGM3), encoded by *Igtp*, is a p47 GTPase that also controls cross-presentation in DCs. IRGM3-deficient DCs were proved to exhibit a major impairment in their ability to cross-present phagocytozed antigens to CD8^+^ T cells (80). Chemokine (C–C motif) ligand 3 and 4, encoded by *Ccl3* and *Ccl4*, respectively, are produced at the immunological synapse between DCs and T cells and increase the chance for migrating CCR5-expressing CD8^+^ T cells to contact DCs by a factor of 2–4 (81). Furthermore, upregulation of *Dhx58* suggested the presence of LPG2, a RIG-I-like receptor that is required for controlling antigen-specific CD8^+^ T cell survival and fitness during peripheral T cell number expansion in response to virus infection (82), while upregulation of *Tnfsf8* indicated the expression of CD135 which is the ligand for CD30 whose signaling plays important role in the generation of long-lived memory CD8^+^ T cells (83). *Il15* and *Il15ra* were also found exclusively upregulated in DCs stimulated with mix B. Interestingly, previous studies have shown that coordinate expression of IL-15 and IL-15Rα by the same accessory cells such as DCs is required for supporting both NK and CD8^+^ memory T cell homeostasis (84, 85).

As mentioned earlier, CD8^+^ T cell responses are crucial mediators of immunity against intracellular pathogens like *L. infantum* parasites and a protective peptide-based vaccine targeting such immune responses might open a new way toward the battle over VL. Nevertheless, CD4^+^ T_H1_ cells also play a central role in *Leishmania*-specific response and thus they must be taken into account in vaccination strategies against the disease. Both DCs stimulated with the mix B or mix C were characterized by IL-12 production (Figure 5), a pro-inflammatory cytokine with great importance in the activation of T_H1_ cell responses (86), and promoted allogeneic T cell proliferation and IFNγ production by CD4^+^ T cells (Figures 6 and 7), required for the immunity against VL. It can be argued that the nature of nanoformulations has a contribution to this fact, since in a previous study PLGA-based NPs loaded with CpG were proved to induce greater cytokine production and T cell proliferation than the oligonucleotide alone (87). Further, the presence of IL-4-producing CD4^+^ T cells in the same population density as in the case of T cells co-cultured with unstimulated DCs indicated the absence of a T_H2_ response (Figure 7) that is unwilling in the fight against VL.

Microarray data analysis further supported these findings unveiling up- (*Il6, Il12b, Ccl4, Cxcl3, Tnfsf9*, etc.) and downregulated genes (*Icos-l*, etc.) involved in T_H_ cell aggregation and activation (Data Sheet S1 in Supplementary Material; Figures 8 and 9; Table 4). However, the presence of upregulated genes exclusively expressed in DCs stimulated with mix B demonstrated that these specific cells might be in a more advanced state of maturity and functional differentiation in terms of T_H_ polarization than DCs stimulated with mix C (Table 4). For example, a number of upregulated genes involved in the regulation of cytokine production or in the response to cytokine stimulus, such as *Cish, Il1f6, Osm, Cxcl9, Tnfsf15, Ddit3*, and *Dll4*, are considered markers of T cell activation and polarization toward T_H1_ cells. *Cish* is identified as a STAT5 target gene and encodes a cytokine-inducible SH2-containing protein that plays a crucial role in type 1 DCs development (T_H1_ polarization), as well as in DC-mediated cytotoxic T cell activation, since *Cish* knockdown was found to reduce the expression of MHC class I, co-stimulatory molecules and pro-inflammatory cytokines in bone marrow-derived DCs (88). Delta-like 4 (DLL4) is a protein encoded by the *Dll4* gene and its expression by DCs is critical for eliciting T cell responses. Activated DLL4^+^ DCs were more capable to promote T_H1_ and T_H17_ differentiation than unstimulated DCs (89). Furthermore, CXCL9 is a well-known T_H1_ attractant molecule, whereas oncostatin M—encoded by the *Osm* gene—is a pleiotropic cytokine that was found to induce the allogeneic stimulatory capacity of DCs by promoting the production of IL-12 and increase the production of IFNγ by T cells in MLRs that would be expected to contribute to the T_H1_ polarization of the immune response (90). *Il1f6* encodes the cytokine IL36A whose signaling pathway activates DCs and amplifies T_H1_ responses by enhancing proliferation and T_H1_ polarization of naïve CD4^+^ T cells (91).

The *in vitro* screening promoted the peptide-based PLGA nanoformulations with MPLA incorporation as a promising nanovaccine candidate and, therefore, immunization of HLA A2.1 transgenic mice was performed to unveil the induction of peptide-specific CD8^+^ T cell responses. Results obtained confirmed the ability of PLGA nanoformulations with MPLA incorporation to target efficiently DCs, promoting peptide presentation through MHC class I molecules and thus inducing peptide-specific IFNγ-producing CD8^+^ T cells (Figure 10). This finding provides evidence that the peptide-specific response is attributed to the HLA A*0201 epitopes predicted by the *in silico* analysis and included in the chimeric peptides. According to the *in silico* analysis chimeric peptides also contain H2-Db restricted epitopes that might impact the TCR repertoire and further investigation is required. However, a study, focused on the use of this strain of transgenic mice as a model of human immune responses, revealed an extended epitope overlap in human Dryvax vaccines expressing HLA A*0201 and HLA A2.1 transgenic mice (92). In a previous study of our group, a single peptide of CPA (CPA_p2) co-encapsulated with MPLA in PLGA NPs induced CPA_p2-specific cellular and humoral immune responses and conferred acute protection against *L*. *infantum* infection in BALB/c mice, mediated by IFNγ-producing CD8^+^ T cells (93). Encapsulating more than one and longer synthetic peptides derived from three different immunogenic *Leishmania* proteins, more intense CD8^+^ T cell responses were achieved, required in the immunity developed against VL. This tactics is in agreement with other studies underlining that vaccines designed to address a broad range of specificities are capable of inducing polyclonal effector T cells promoting protection (51, 94, 95).

Protection assays conducted in HLA A2.1 transgenic mice confirmed that this tactics could be considered as an improved strategy in terms of prophylactic efficacy against *L. infantum* infection (Figure 11). HLA A2.1 transgenic mice are created on a C57BL/6 background characterized by susceptibility to *L. infantum* infection with a cure profile on the infection outcome (96). The selection of the specific time points for the estimation of immunization’s protective effect was based on the infection kinetics in these mice. According to the time course of infection, the parasite burden reached a peak at 1 month post-infection in the liver and at 2 months post-infection in the spleen, followed by a decrease in both visceral organs. The results obtained from the limiting dilution assay revealed a significant reduction in hepatic and splenic parasite burden at 1 month post-infection by 72.81 and 61.98%, respectively, compared to the non-immunized control group. It is of particular interest that this protective effect was preserved at 2 months post-infection in both visceral organs (64.4 and 73.64% reduction in liver and spleen, respectively) indicating that immunization could accelerate the self-curing profile. The above findings combined with the fact that mice immunized with PLGA-MPLA nanoformulations exhibited comparable levels of parasite burden with the non-immunized mice provide evidence that the peptide-based PLGA nanoformulations with MPLA incorporation conferred a significant peptide-specific protection against *L. infantum* infection that was maintained 2 months post-infection before starting the self-curing phase on the infection outcome.

Conclusively, our findings supported that the encapsulation of more than one chimeric multi-epitope peptides from different immunogenic *L. infantum* proteins in a proper biocompatible delivery system with the right adjuvant is considered as an improved promising approach for the development of a vaccine against VL, since it induced a strong maturation profile in DCs and enabled them to present efficiently the pathogen-derived peptides to T cells inducing peptide-specific CD8^+^ T cells with IFNγ production and conferring significant protection against *L. infantum* infection.

## Ethics Statement

This study was carried out in accordance with the recommendations of National Law 2013/56 and the EU Directive 2010/63/EU for animal experiments and complied with the ARRIVE guidelines. The protocol was approved by the institutional Animal Bioethics Committee.

## Author Contributions

Conceived and designed the experiments: EK. Performed the experiments: EA, MA, and OK. Performed the bioinformatics analysis of the microarray data: ST, AH, and EA. Analyzed the data: EA, MA, ST, OK, AH, CK, and EK. Wrote the paper: EA and EK. All the authors critically revised the work, approved the version to be published, and agreed to be accountable for its content.

## Conflict of Interest Statement

The authors declare that the research was conducted in the absence of any commercial or financial relationships that could be construed as a potential conflict of interest.
