# Supplementary material for: A Poly(Lactic-co-Glycolic) Acid Nanovaccine Based on Chimeric Peptides from Different Leishmania infantum Proteins Induces Dendritic Cells Maturation and Promotes Peptide-Specific IFNγ-Producing CD8+ T Cells Essential for the Protection against Experimental Visceral Leishmaniasis
Source: Front Immunol. 2017 Jun 13;8:684. doi: 10.3389/fimmu.2017.00684 (PMC5468442; doi:10.3389/fimmu.2017.00684)
Supplement: Supplementary file 1 [file Data_Sheet_1.DOCX]

Supplementary Material

A Poly(Lactic-*co*-Glycolic) Acid Nanovaccine Based on Chimeric Peptides from Different *Leishmania* *infantum* Proteins Induces Dendritic Cells Maturation and Promotes Peptide-Specific IFNγ-Producing CD8^+^ T Cells Essential for the Protection against Experimental Visceral Leishmaniasis

Evita Athanasiou, Maria Agallou, Spyros Tastsoglou, Olga Kammona, Artemis Hatzigeorgiou, Costas Kiparissides and Evdokia Karagouni^*^

*** Correspondence:**Dr. Evdokia Karagouni
ekaragouni@pasteur.gr

# Supplementary Figures

**Supplementary Figure 1. Encapsulation of chimeric peptides in poly(lactic-*co*-glycolic) acid (PLGA) nanoparticles (NPs) induced a strong maturation profile in dendritic cells (DCs) from HLA A2.1 transgenic mice.** The diagrams demonstrate the effect of chimeric peptide’s encapsulation in PLGA NPs on DCs maturation in terms of CD40, CD80, CD86, MHC class I and II molecules expression. DCs were stimulated for 24 h with PLGA-chCPAp (A), PLGA-chH1p (B) and PLGA-KMP-11p (C) nanoformulations or with each chimeric peptide in a soluble form, stained against surface markers and analyzed with flow cytometry. DCs cultured in medium alone or in the presence of 1 µg/ml LPS were used as negative and positive control, respectively. Results are expressed as the mean±SD of three independent experiments. Significant differences between DCs stimulated with the peptide-based PLGA nanoformulations and DCs stimulated with chimeric peptides in soluble form are indicated by * (p<0.05), ** (p<0.01), or *** (p<0.001).

**Supplementary Figure 2. Monophosphoryl lipid A (MPLA) incorporation in peptide-based poly(lactic-*co*-glycolic) acid (PLGA) nanoformulations induced a strong maturation profile in dendritic cells (DCs) from HLA A2.1 transgenic mice.** The diagrams demonstrate the effect of MPLA adjuvant incorporation in peptide-based PLGA nanoformulations on DCs maturation in terms of CD40, CD80, CD86, MHC class I and II molecules expression. DCs were stimulated for 24 h with PLGA-chCPAp-MPLA (A), PLGA-chH1p-MPLA (B) and PLGA-KMP-11p-MPLA (C) nanoformulations or with each chimeric peptide plus MPLA in soluble form, stained against surface markers and analyzed with flow cytometry. Significant differences between DCs stimulated with the peptide-based PLGA nanoformulations having incorporated MPLA and DCs stimulated with the peptide-based PLGA nanoformulations having encapsulated each chimeric peptide alone are indicated by * (p<0.05), ** (p<0.01), or *** (p<0.001).

**Supplementary Figure 3. Surface modification with p8 in peptide-based poly(lactic-*co*-glycolic) acid (PLGA) nanoformulations also induced a strong maturation profile in dendritic cells (DCs) from HLA A2.1 transgenic mice.** The diagrams demonstrate the effect of surface modification with p8 on DCs maturation in terms of CD40, CD80, CD86, MHC class I and II molecules expression. DCs were stimulated for 24 h with p8-PLGA-chCPAp (A), p8-PLGA-chH1p (B) and p8-PLGA-KMP-11p (C) nanoformulations, stained against surface markers and analyzed with flow cytometry. Results are expressed as the mean±SD of three independent experiments. Significant differences between DCs stimulated with the surface modified peptide-based PLGA nanoformulations and DCs stimulated with the peptide-based PLGA nanoformulations having encapsulated each chimeric peptide alone are indicated by * (p<0.05), ** (p<0.01), or *** (p<0.001).
