# Supplementary figures and images for: A Poly(Lactic-co-Glycolic) Acid Nanovaccine Based on Chimeric Peptides from Different Leishmania infantum Proteins Induces Dendritic Cells Maturation and Promotes Peptide-Specific IFNγ-Producing CD8+ T Cells Essential for the Protection against Experimental Visceral Leishmaniasis
Source: Front Immunol. 2017 Jun 13;8:684. doi: 10.3389/fimmu.2017.00684 (PMC5468442; doi:10.3389/fimmu.2017.00684)

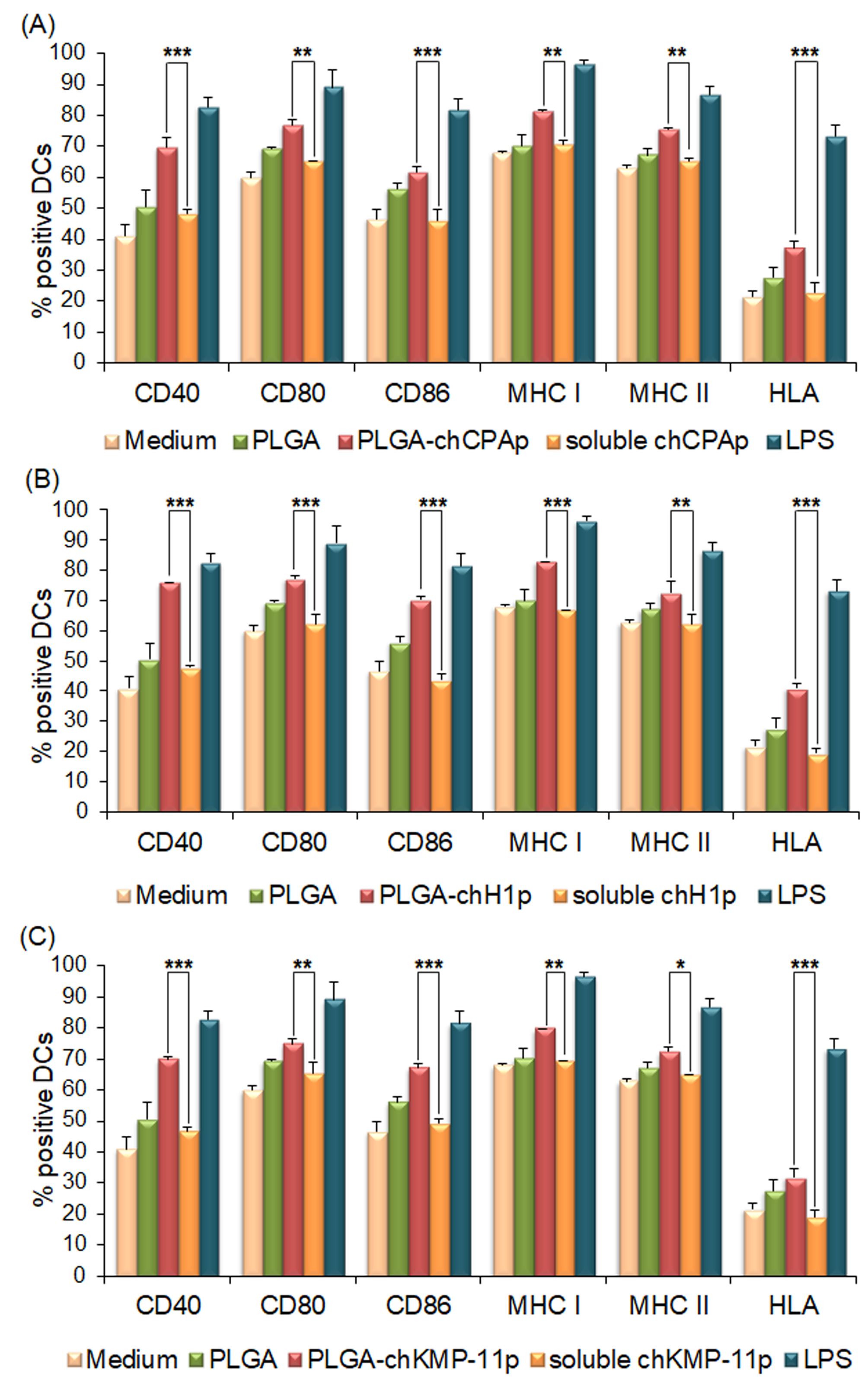

Supplement: Supplementary file 3 [file Image_1.TIF]

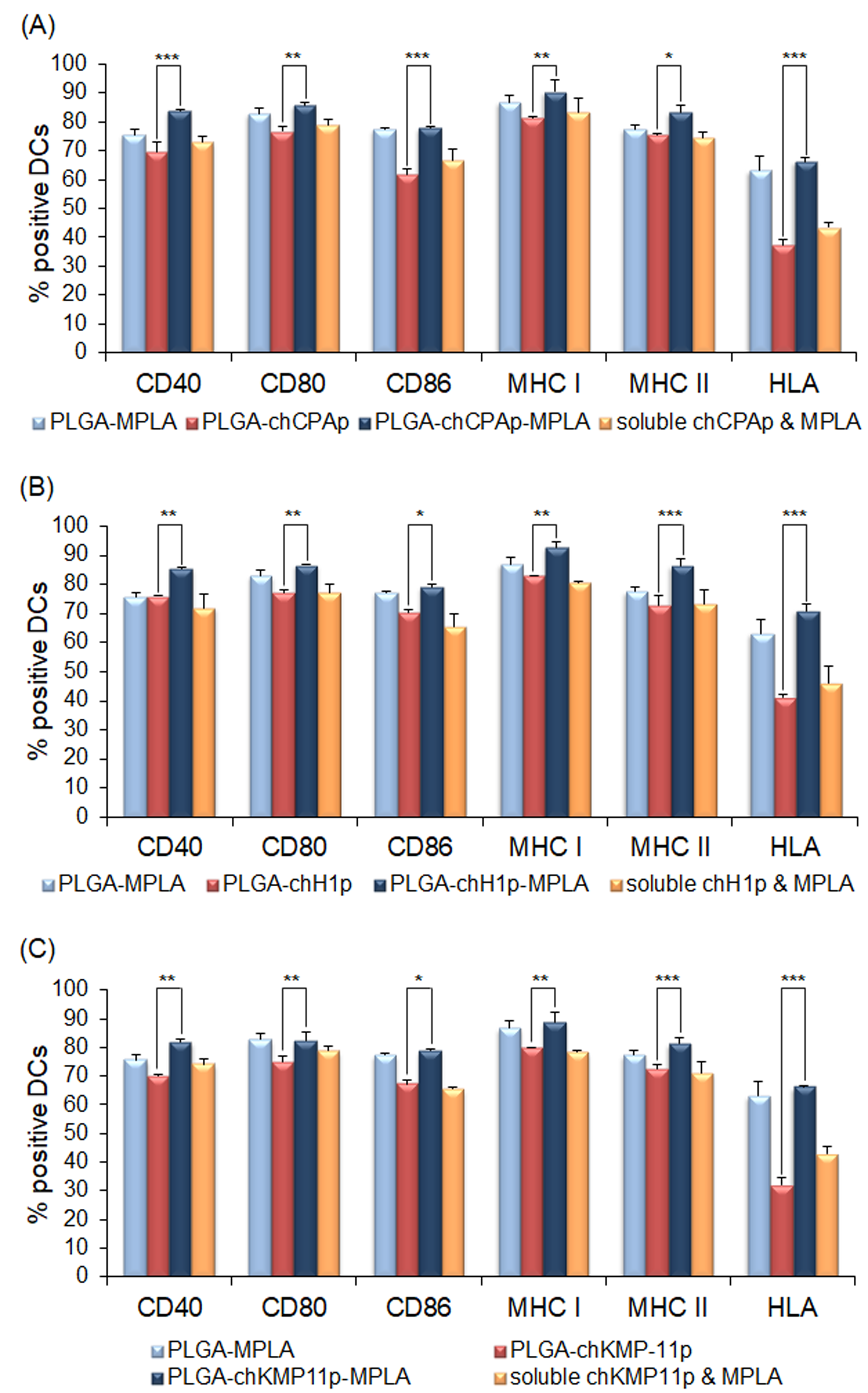

Supplement: Supplementary file 4 [file Image_2.TIF]

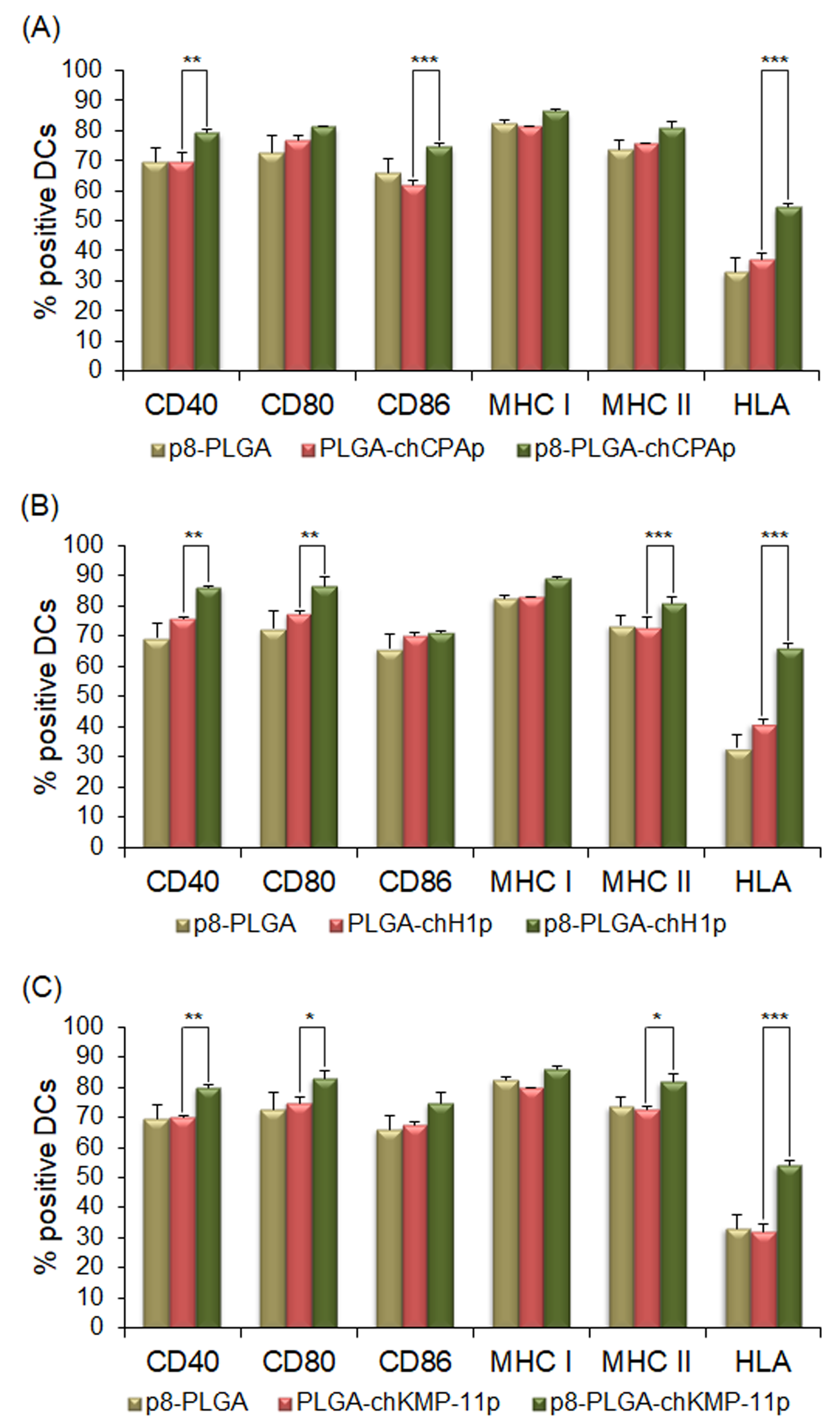

Supplement: Supplementary file 5 [file Image_3.TIF]
